# Evaluating genomic data for management of local adaptation in a changing climate: A lodgepole pine case study

**DOI:** 10.1111/eva.12871

**Published:** 2019-09-30

**Authors:** Colin R. Mahony, Ian R. MacLachlan, Brandon M. Lind, Jeremy B. Yoder, Tongli Wang, Sally N. Aitken

**Affiliations:** ^1^ Centre for Forest Conservation Genetics and Department of Forest and Conservation Sciences University of British Columbia Vancouver BC Canada; ^2^ Department of Ecology and Evolutionary Biology Yale University New Haven CT USA; ^3^ Department of Biology California State University Northridge Northridge CA USA

**Keywords:** assisted gene flow, climate change adaptation, ecological genetics, genomic variation, landscape genomics, phenotypic variation

## Abstract

We evaluate genomic data, relative to phenotypic and climatic data, as a basis for assisted gene flow and genetic conservation. Using a seedling common garden trial of 281 lodgepole pine (*Pinus contorta*) populations from across western Canada, we compare genomic data to phenotypic and climatic data to assess their effectiveness in characterizing the climatic drivers and spatial scale of local adaptation in this species. We find that phenotype‐associated loci are equivalent or slightly superior to climate data for describing local adaptation in seedling traits, but that climate data are superior to genomic data that have not been selected for phenotypic associations. We also find agreement between the climate variables associated with genomic variation and with 20‐year heights from a long‐term provenance trial, suggesting that genomic data may be a viable option for identifying climatic drivers of local adaptation where phenotypic data are unavailable. Genetic clines associated with the experimental traits occur at broad spatial scales, suggesting that standing variation of adaptive alleles for this and similar species does not require management at scales finer than those indicated by phenotypic data. This study demonstrates that genomic data are most useful when paired with phenotypic data, but can also fill some of the traditional roles of phenotypic data in management of species for which phenotypic trials are not feasible.

## 1 INTRODUCTION

Climate change is impacting the forests of western North America through tree injury and mortality from droughts, floods, wildfires, disease, and insect outbreaks (Allen et al., 2010; Anderegg et al., 2015; Buotte et al., 2018; van Mantgem et al., 2009; McDowell & Allen, 2015; Reyer, Rammig, Brouwers, & Lnagerwisch, 2015). There is also mounting evidence that changes in climate are disrupting local adaptation in plants (Mcgraw et al., 2015; Wilczek et al., 2019), with impacts to productivity of long‐lived tree species (Leites, Robinson, Rehfeldt, Marshall, & Crookston, 2012; Rehfeldt, Ying, Spittlehouse, & Hamilton, 1999) and conservation status of vulnerable species (Parmesan, 2006). In response, forest managers are seeking guidance on which source populations to use for planting, as the long‐practiced “local is best” strategy no longer matches trees with the climates to which they are adapted (Aitken & Bemmels, 2016). There is also a need to characterize the spatial scale and genetic structure of local adaptation to understand the capacity of populations to adapt to climate change without human intervention (Kawecki, 2008; Kremer et al., 2012; McKenney, Pedlar, Lawrence, Campbell, & Hutchinson, 2007). These efforts are supported by long‐term provenance trials for some commercial tree species such as lodgepole pine (*Pinus contorta* Dougl. ex Loud), which accounts for 25% of the approximately 580 million seedlings planted in Canada each year (Canadian Forest Service, 2019). However, the cost and duration of comprehensive provenance trials has been prohibitive for many tree species of commercial and conservation importance, and rapid assessment is needed for climate change adaptation (Aitken, Yeaman, Holliday, Wang, & Curtis‐McLane, 2008). A comparison of alternative genecological data sources in a well‐studied species like lodgepole pine can inform the use of these methods in other species.

For centuries, local adaptation has been quantified and managed in tree species using phenotypic data from long‐term provenance trials and short‐term common gardens (Langlet, 1971). In the past two decades, climate data have been used to extend phenotypic inferences of local adaptation across landscapes and to project mismatches between adaptive variation and future climates (e.g., St. Clair & Howe, 2007; Wang, O'Neill, & Aitken, 2010). Genomic data are now widely used as a third source of insight into local adaptation for nonmodel species (e.g., Exposito‐Alonso et al., 2018; Fitzpatrick & Keller, 2015; Sork et al., 2013; Wadgymar et al., 2017). While the genomic basis of local adaptation has been extensively studied (Li et al., 2017; Sork, 2018), urgently needed applications of genomic data to mitigate effects of climate change are in their infancy (Shafer et al., 2015). These applications can be advanced by understanding the ways in which genomic data complement and overlap with phenotypic and climatic data in characterizing local adaptation.

For most tree populations, the capacity to track suitable climates via migration and establishment will be outpaced by the rate of climate change (Davis & Shaw, 2001; Gray & Hamann, 2013; McLachlan, Clark, & Manos, 2005), with implications for the health and productivity of wild forests and those planted for wood or carbon sequestration. Assisted gene flow (AGF), the “intentional translocation of individuals within a species range to facilitate adaptation to anticipated local conditions” (Aitken & Whitlock, 2013), is a strategy for mitigating these deleterious effects of mismatches between genotypes and climate. For instance, populations adapted to warmer locations of the species' range are faster growing, although less cold hardy, for many temperate and boreal species (Aitken & Bemmels, 2016; Wang et al., 2010). If genotypes are moved into suitable climates, but not so far that they suffer from cold injury or other types of maladaptation, this faster growth rate is expected to translate into higher survival, better health, and greater productivity (e.g., Wadgymar, Cumming, & Weis, 2015). When the motivation for planting is conservation, AGF could bolster the demographics and genetic diversity of populations of rare species or accelerate stand development for habitat and other ecosystem services (Kelly & Phillips, 2016; Lunt et al., 2013).

The imperative for AGF with forest trees is acute not only due to their economic and ecological value, but also due to the high rate of climate change they experience per generation (Aitken et al., 2008; Alberto et al., 2013; McLachlan et al., 2005; Petit & Hampe, 2006). Fortunately, the feasibility of AGF in forest trees is high due to (a) the long history of study and understanding of local adaptation to climate in many widespread species (Langlet, 1971; Morgenstern, 1996); (b) the infrastructure and operational practices that already exist for collecting or producing seeds, growing seedlings, and reforesting harvested or otherwise disturbed areas (Aitken & Bemmels, 2016); and (c) the general lack of strong population structure and isolation that might lead to outbreeding depression (Howe et al., 2003; Mitton & Williams, 2006; Neale & Savolainen, 2004; Savolainen, Pyhäjärvi, & Knurr, 2007). For example, Yeaman et al. (2016) report weak genetic differentiation (*F*_ST_ = 0.016) among western Canadian lodgepole pine populations.

Effective AGF strategies require an understanding of the major climatic drivers of local adaptation and how strongly populations are differentiated along these climatic gradients. Forest scientists have traditionally used provenance trials—in situ field‐based common garden experiments that usually involve partial reciprocal transplants—to understand links between phenotypes under divergent selection and the environments driving those differences (Langlet, 1971; Lind, Menon, Bolte, Faske, & Eckert, 2018; Morgenstern, 1996). Long‐term provenance trials allow researchers to disentangle the genetic and climatic controls on fitness‐related traits such as survival and growth. Further, dendrochronological studies of provenance trials can retrospectively identify population responses to climatic variability such as frost and drought (e.g., Isaac‐Renton et al., 2018; Montwé, Isaac‐Renton, Hamann, & Spiecker, 2018). However, provenance trials are unfeasible for many species due to the decades‐long time frame needed to obtain meaningful data, by the restricted geographic and climatic scopes of both provenances and planting sites for many existing trials (Aitken et al., 2008; Kawecki & Ebert, 2004; de Villemereuil, Gaggiotti, Mouterde, & Till‐Bottraud, 2015), and the resources required for new experiments, including suitable planting sites and adequate seed collections (Blanquart, Kaltz, Nuismer, & Gandon, 2013; Flanagan, Forester, Latch, Aitken, & Hoban, 2018; Morgenstern, 1996).

Seedling common gardens are complementary to traditional provenance trials in several ways. Single‐environment seedling common gardens can be used to quantify phenotypic differentiation among populations and to develop transfer functions (Matyas, 1994; O'Neill, Hamann, & Wang, 2008), while multiple‐environment experiments can be used to test for environmental forces driving this differentiation. Such experiments allow for detailed phenotyping of climate‐related traits at vulnerable seedling stages that have important fitness consequences for the populations under consideration (e.g., phenology, cold or drought hardiness, growth, and allocation of biomass; Savolainen et al., 2007, Alberto et al., 2013, and Lind et al., 2018).

Patterns of phenotypic variation among populations were traditionally described in geographic terms, but the advent of high‐resolution gridded climate data (e.g., PRISM, Daly, Neilson, & Phillips, 1994), has allowed more precise inferences of the spatial patterns of local adaptation (Wang et al., 2010) and has facilitated the transition from geography‐based to climate‐based seed transfer (O'Neill et al., 2017). When integrated with climate change projections (e.g., ClimateNA, Wang, Hamann, Spittlehouse, & Carroll, 2016; and WorldClim, Fick & Hijmans, 2017), climate data provide the essential basis for AGF and address some of the shortcomings of geographically based seed zones. While generic approaches to climate variable selection may provide a first approximation for AGF (e.g., niche modeling), information tailored to species‐specific patterns relating adaptive variation to climate will better tailor AGF strategies (e.g., O'Neill et al., 2017), as climatic factors limiting a species' niche may not be those driving differentiation among populations.

In situations where phenotypic data are unavailable or limited, genomic data could be useful for inferring patterns of adaptive differentiation among populations and climatic drivers of local adaptation. Population genomic approaches for detecting adaptive variation have become feasible within the last decade (reviewed in Sork et al., 2013, Prunier, Verta, & MacKay, 2015, and Lind et al., 2018). Next‐generation sequencing methods now allow for the genotyping of large numbers of variants (e.g., single nucleotide polymorphisms, SNPs) in nonmodel species to inform management and conservation decisions (Flanagan et al., 2018; Lotterhos, Yeaman, Degner, Aitken, & Hodgins, 2018; Rellstab, Dauphin, Zoller, Brodbeck, & Gugerli, 2019). Genotype–environment association (GEA) approaches can identify both the environmental drivers of local adaptation and loci underlying locally adaptive traits (De Mita et al., 2013; Rellstab, Gugerli, Eckert, Hancock, & Holdregger, 2015; Schoville et al., 2012). Likewise, genotype–phenotype association (GPA) studies can identify loci associated with adaptive phenotypes (Holliday et al., 2017; Neale & Savolainen, 2004; Prunier et al., 2015). These methods can be combined to identify suites of potentially locally adapted loci (e.g., Yeaman et al., 2016). Despite the extensive literature on genomic approaches for characterizing local adaptation, and the potential for these approaches to generate knowledge of local adaptation more quickly than provenance trials, we are not aware of any operational uses of genomic data to guide seed transfer or AGF. Genomic data can also provide unique insights into local adaptation that are not available from phenotypic or climatic data alone. For example, rangewide phenotypic clines can potentially mask more localized allelic clines that underlie adaptive traits (see Box 1). Similarly, the spatial structure of standing variation in adaptive alleles—an important consideration for AGF and in situ genetic conservation—can only be inferred from genomic data.

Box 1 **The structure of allelic variation underlying phenotypic clines in adaptive traits**

For widespread tree species that experience both strong diversifying selection and high gene flow, climatic gradients often drive clinal variation in phenotypes (Alberto et al., 2013; Endler, 1977). However, the number and geographic distribution of adaptive loci underlying these patterns is, for the most part, unknown.

**Figure 1:**
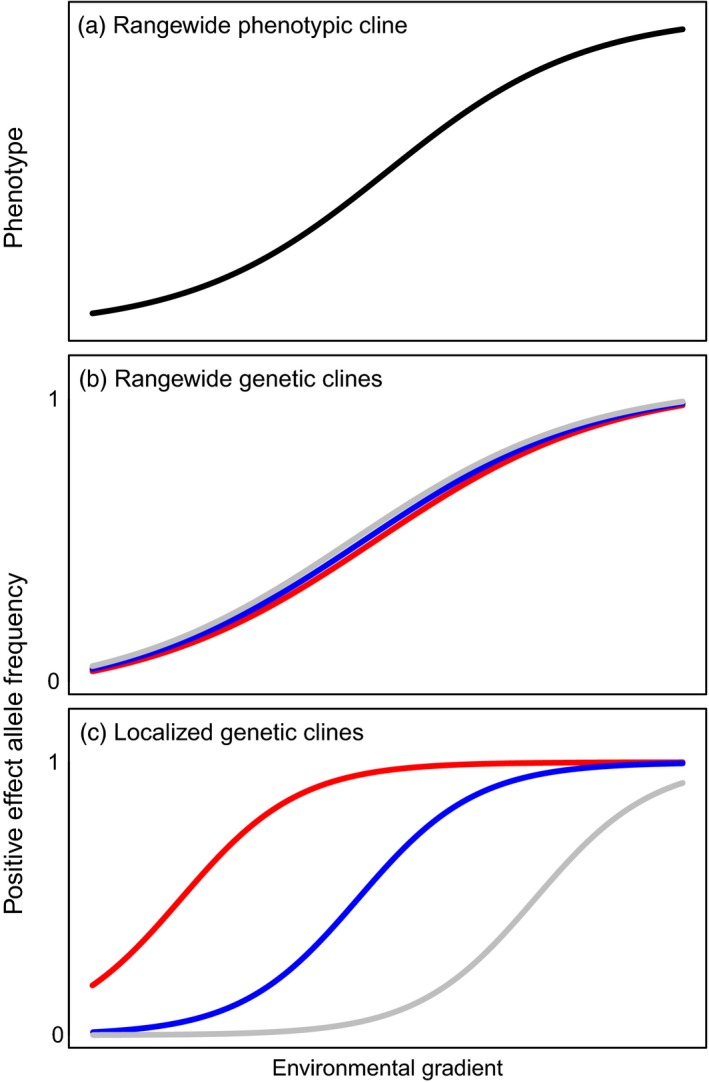
Illustration of rangewide versus localized genetic clines (b, c) underlying a continuous phenotypic cline (a) along an environmental gradient (after Barton, 1999)

There are two ways for genetic clines to produce a rangewide cline in an additive polygenic trait (Figure 1). The first is to have concordant clines in the underlying loci, representing a gradual rangewide shift in allelic frequency across all underlying loci (Figure 1b) that therefore matches the rangewide phenotypic cline (Figure 1a). Alternatively, a phenotypic cline can result from multiple distinct, localized genetic clines, each providing variation sequentially over short sections of the environmental gradient (Barton, 1999; see also Box 3 in Savolainen et al., 2007), as depicted in Figure 1c.

The degree to which local adaptation is structured as localized, sequential genetic clines has implications for AGF, as this may reduce the amount of standing adaptive variation and thus adaptive potential. Ultimately, the spatial scale of adaptation is a function of gene flow, selection, and drift. In species with long‐isolated populations and little gene flow, such structure could also risk lower compatibility between native and transplanted individuals, but outbreeding depression is unlikely in widespread, abundant, wind‐pollinated trees (Aitken & Whitlock, 2013). If adaptive variation is distributed as concordant rangewide genetic clines, loci underlying an adaptive trait will be polymorphic throughout most of the species range, except perhaps at the range margins, or in otherwise isolated or small populations. In this case, standing variation should exist for adaptive loci that could enable in situ adaptation to climatic change, as long as locally novel climatic conditions exist elsewhere in the species range and are not isolated from gene flow. Localized clines, in contrast, imply that standing variation in a subset of adaptive alleles is limited to only a portion of the species' range.

The objective of this study is to evaluate genomic data, relative to phenotypic and climatic data, as a basis for assisted gene flow and genetic conservation of locally adapted conifers. We address three research questions using phenotypic and genomic data from 281 western Canadian populations of lodgepole pine. Firstly, *what is the relative value of genomic data *versus* climatic and geographic data in explaining locally adaptive phenotypic variation?* We address this question by comparing the proportion of variance in four seedling traits that can be explained by geographic, climatic, and several types of genomic data including a full SNP array, a large set of control markers, and loci inferred from both genotype–phenotype associations (GPAs) and genotype–environment associations (GEAs). Secondly, *can genomic data identify the climatic drivers of local adaptation?* We use phenotypic data from both a short‐term seedling common garden study and a long‐term provenance trial to contrast the predicted importance of various climatic drivers of phenotypic differentiation to that predicted from genomic data (GEA loci). Thirdly, we examine information that is uniquely available from genomic data—the genetic clines underlying phenotypic clines—to address the question: *what is the spatial scale of local adaptation to climate?* These assessments identify the contributions that genomic data can make to assisted gene flow and genetic conservation in a changing climate.

## 2 METHODS

### 2.1 Phenotypic data

#### 2.1.1 Seedling common garden experiment

**Figure 2:**
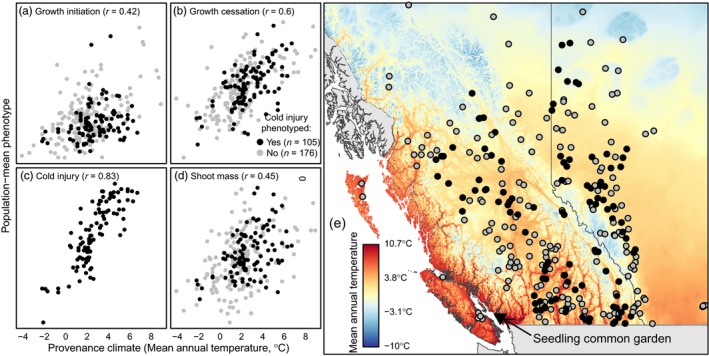
Phenotypic clines of four traits in lodgepole pine seedlings grown in the Vancouver common garden**.** A total of 1,594 seedlings from 281 provenances across British Columbia and Alberta, Canada (gray and black circles), were phenotyped for growth initiation (a), growth cessation (b), and 3‐year shoot mass (d). A subset of 922 seedlings from 105 provenances (black circles) were tested for autumn cold injury (c). Phenotypic clines (a–d) are plotted on an environmental gradient of mean annual temperature, mapped in (e)

The primary phenotypic data in this study originate from a raised bed common garden of 1,594 lodgepole pine seedlings at Totem Field at the University of British Columbia in Vancouver, BC. Design, establishment, and measurement of the common garden, summarized here, are described in detail by MacLachlan, Wang, Hamann, Smets, and Aitken (2017). Briefly, seedlots originated from 281 provenances representing lodgepole pine's climatic range within British Columbia and Alberta (Figure 2e and Figure S13). Seedlots were predominantly selected from the range of the Rocky Mountain subspecies (*P. contorta* Dougl. ex Loud. ssp. *latifolia* [Engelm.] Critchfield), but also include the coastal subspecies (*P. contorta* Dougl. ex Loud. ssp. *contorta*) and the region of hybridization with jack pine (*Pinus banksiana* Lamb.) in northern Alberta.

Our study utilizes phenotypic data from four traits: growth initiation, growth cessation, autumn cold injury, and shoot mass (methods in MacLachlan et al., 2017). We removed experimental effects from phenotypic values by reporting phenotypes as *z*‐standardized residuals of a linear mixed‐effects model, implemented with ASreml‐R (Butler, 2009), in which experimental block and location within block are random effects:

(1) $$Y_{\mathrm{ijk}}=\mu +B_{j}+L{\left(B\right)}_{\mathrm{jk}}+e_{\mathrm{ijk}}$$

where *Y_ijk_* is the phenotypic observation of a trait made on individual *i* grown in the *j*th block (*B*), at the *k*th seedling location (*L*) nested within block (*L*(*B*)*_jk_*), *μ* is the experimental mean, and *e* is the residual error of individual *i*.

#### 2.1.2 Illingworth provenance trial

We analyzed 20‐year heights from the Illingworth lodgepole pine provenance trial to corroborate the inferences from the Vancouver seedling common garden with longer‐term data from sites more typical for this species. This trial, established in 1974 by the BC Ministry of Forests (Illingworth, 1978; Wang et al., 2010), tested a rangewide (New Mexico to Yukon) collection of 140 provenances at 60 sites in interior British Columbia. We assessed the strength of the univariate relationships between population‐mean 20‐year height and provenance climate for three contrasting trial sites: one each from southern (PETI), central (NILK), and northern (WATS) British Columbia (Figure S1). We estimated an adjusted *R* ^2^ for the quadratic relationship between provenance climate and the average 20‐year heights of the populations at each test site. We estimated this relationship for each of the 19 standard climate variables used in this study (Table 1). Reported results are the mean *R* ^2^ over the three sites.

**Table 1:** The set of 19 bioclimatic variables used in this study

Environmental Variable (unit)	Abbreviation
Mean annual temperature (°C)	MAT
Mean warmest month temperature (°C)	MWMT
Mean coldest month temperature (°C)	MCMT
Continentality (MWMT minus MCMT) (°C)	TD
Mean annual precipitation (mm)	MAP
May to September precipitation (mm)	MSP
Annual heat:moisture index (MAT + 10)/(MAP/1,000)) (°C/μm)	AHM
Summer heat:moisture index ((MWMT)/(MSP/1000)) (°C/μm)	SHM
Degree‐days below 0°C, chilling degree‐days	DD_0
Degree‐days above 5°C, growing degree‐days	DD5
Number of frost‐free days (days)	NFFD
Frost‐free period (days)	FFP
The day of the year on which FFP begins (Julian date)	bFFP
The day of the year on which FFP ends (Julian date)	eFFP
Precipitation as snow between August and July (mm)	PAS
Extreme minimum temperature over 30 years (°C)	EMT
Extreme maximum temperature over 30 years (°C)	EXT
Hargreaves reference evaporation (mm)	Eref
Hargreaves climatic moisture deficit (mm)	CMD

### 2.2 Climate data

Climate normals for the 1961–1990 period for each provenance in the seedling common garden were obtained from ClimateNA (Wang et al., 2016), using the latitude, longitude, and elevation of each seedlot. The 19 bioclimatic variables used in this study (Table 1) are the same as used in previous analyses of genomic datasets from the AdapTree Project, selected *a priori* based on relevance to the species biology and environmental variation across provenances (Lotterhos et al., 2018; MacLachlan et al., 2017; Yeaman et al., 2016). These variables are not filtered for collinearity, and several variable pairs are highly correlated (Table S2).

### 2.3 Genomic data

#### 2.3.1 SNP table

DNA was extracted from tissue of spring needles using a Macherey‐Nagel Nucleospin 96 Plant II Core™ Kit, automated on an Eppendorf EpMotion 5075™ liquid handling platform. Samples were genotyped by Neogen GeneSeek (Lincoln, Nebraska) using the AdapTree lodgepole pine Affymetrix Axiom 50K lodgepole pine SNP array. SNP discovery for this array was based on the lodgepole pine sequence capture dataset described by Suren et al. (2016). It included probes for the exons of 24,388 genes, as well as intergenic regions, with intron–exon boundaries identified by mapping the lodgepole pine transcriptome to the loblolly pine (*Pinus taeda* L.) v1.01 draft genome (Neale et al., 2014; Zimin et al., 2014). SNPs were selected for inclusion based on preliminary GEA and GPA analyses using phenotypes for seedling traits (Yeaman et al., 2016), differentially expressed genes (Yeaman et al., 2014), candidate genes for climate adaptation from other conifers, mappable SNPs for a linkage map, and a set of randomly selected intergenic SNPs to control for population structure. Genotypes from the SNP table were filtered to retain 36,384 SNPs with a minor allele frequency ≥0.01. Of these filtered loci, 3,934 were intergenic control SNPs for population structure correction in association analyses. Excluding this “control set,” the final candidate adaptive SNP table used in associations contained 32,407 SNPs. We genotyped 1,594 seedlings from the Vancouver outdoor seedling common garden and an additional 1,906 seedlings from the same 281 provenances grown in a separate growth chamber experiment (Liepe, Hamann, Smets, Fitzpatrick, & Aitken, 2016), for a total median sample size of 11 seedlings (range seven to 24) for each provenance (Figure S2).

#### 2.3.2 Genotype–Phenotype Association (GPA)

We implemented GPA using the phenotypic residual values (from Equation 1) for each of the four traits measured at the Vancouver outdoor seedling common garden using the linear regression‐based *mlma* function in GCTA (Yang, Lee, Goddard, & Visscher, 2011). We corrected for population structure using the *grm* option of *mlma* with the 3,934 control SNPs described in Section 2.3.1. The number of SNPs per contig ranged from one to 25. For contigs with more than one SNP, we retained the SNP that had the strongest trait association (lowest GCTA *p*‐value) to reduce redundancies due to physical linkage. This reduced the number of available SNPs from 32,407 to 18,525 SNPs. SNPs in the bottom 1% of GPA *p*‐values for each trait were identified as candidate SNPs (*n* = 186 SNPs per trait). For each candidate SNP, the allele that increased the value of a phenotype—called the positive effect allele (PEA)—was identified from the regression slope in the GCTA *mlma* output.

#### 2.3.3 Genotype–Environment Association (GEA)

We used bayenv2 (Coop, Witonsky, Rienzo, & Pritchard, 2010; Günther & Coop, 2013) to identify loci with evidence for responses to environmental selection. The covariance matrix of population structure was estimated by averaging three independent runs, each using the control set of loci for 120,000 iterations. For each centered and standardized environmental variable (Table 1), we ran bayenv2 in test mode for one million iterations across three independent chains for the 32,407 loci using the covariance matrix from the control set (3,934 SNPs) to correct for population structure. To reduce the marker set to one SNP per contig (18,525 SNPs), we retained the SNP from each contig that had the greatest evidence for environmental response (average rank across absolute *rho* and Bayes factor [BF] across the three chains; i.e., six values). To ensure we isolated only loci with the strongest evidence for environmental influence, we re‐ranked these 18,525 loci and retained only those that met two criteria for a given environmental association: (a) The locus was in the top 300 ranked loci for BF for each of the three chains, and (b) it was also in the top 300 ranked loci for absolute value of *rho* for each of the three chains. In addition to using these GEA loci toward our objectives, we report the number of loci identified using these criteria, as well as the overlap between GPA and GEA.

### 2.4 Analyses

We present three analyses that correspond to the three research questions posed in the final paragraph of the Introduction.

#### 2.4.1 Phenotypic variation explained by geographic, climatic, and genomic data

One way of assessing the relative value of geographic, climatic, and genomic data for guiding assisted gene flow and other climate adaptation strategies is to measure the degree to which they can be used to statistically explain locally adaptive phenotypic variation. The dimensionality of the information in each data source is expected to differ: For example, genomewide data may be distributed over many more modes of variation than the three dimensions (latitude, longitude, and elevation) required to fully describe geographic location. These data sources can be compared on more equal terms by extracting their principal components (PCs) and assessing the cumulative explanatory content of increasing numbers of PCs as predictor variables. Explanatory content in this case is measured as proportion of variance explained (*R* ^2^) by a regression of phenotypic values (the response variable) against the PCs of the geographic, climatic, or genomic data (the predictor variables). We used multiple linear regression for this purpose and report the mean *R* ^2^ of a fivefold cross‐validation implemented with the *cv.lm* function of the DAAG package in R (R Core Team, 2017). For comparison, we also performed this analysis with Random Forest regression, a regression tree ensemble learning algorithm that provides cross‐validated modeling of nonlinear relationships and variable interactions (Breiman, 2001). We selected a subset of *climate‐associated* GPA loci with *R* ^2^ > 0.35 in multiple linear regressions on the first five principal components of the 19 climate variables specified in Table 1. The threshold of *R* ^2^ > 0.35 corresponds to the 99.7th percentile of the *R* ^2^ values of the equivalent analysis performed on the control set, as illustrated in Figure S4. To provide a balanced comparison of the control set and GPA set, we performed a separate rarefaction analysis on mutually exclusive random subsamples (*n* = 186 SNPs) of the control set.

#### 2.4.2 Climatic drivers of local adaptation

We examine the congruence of genomic versus phenotypic data in guiding climatic variable selection by contrasting the proportion of variance of individual climate variables that is explained by climate‐associated genomic loci, seedling common garden phenotypes, and long‐term provenance trial phenotypes. For each data source, we conducted one regression for each of the 19 climate variables (Table 1), in which the response (dependent) variable is the provenance climates for a single climate variable. The predictor (independent) variables for the genomic regressions are the first four principal components of the population‐mean minor allele frequencies for the top 300 GEA loci associated with the climate variable of interest (see Section 2.3.3 for GEA methods). The predictor variables for the seedling common garden regressions are the population means of the standardized phenotypes for the four traits (see Section 2.1.1). The predictor variables for the long‐term provenance trial are the 20‐year heights measured at three sites of the Illingworth trial (see Section 2.1.2). Note that the Illingworth data sample a different set of provenances than the genomic and seedling common garden data, and thus are essentially independent of these two other data sources. As in the previous analysis (Section 2.4.1), we used multiple linear regression and report the mean *R* ^2^ of a fivefold cross‐validation for each regression.

#### 2.4.3 Spatial scale of local adaptation to climate

To characterize the genetic clines associated with the traits measured in the seedling common garden, GPA loci were clustered using a Euclidean k‐means algorithm (*kmeans*{*stats*}; R Core Team, 2017). To cluster SNPs, we transposed the population‐mean PEA frequency data so that SNPs occupied the row (observations) position and populations occupied the column (variable) position. Clusters, then, are SNPs that have similar allele frequencies across populations. Similarity in this configuration is distinct from correlation: SNPs with large differences in aggregate allele frequency will be put in separate clusters, even if they are very highly correlated. Hence, this clustering approach is distinct from standard LD clustering approaches based on allele frequency covariance. We use the cluster‐mean PEA frequency for each population to visually summarize the clusters. The mean allelic frequencies of each cluster have reduced variance, due to averaging, which creates artificially smooth (non‐noisy) cluster clines. To restore the variance of the cluster‐mean PEA frequency, we multiplied the cluster‐mean PEA frequency for each population by the mean standard deviation of the SNPs in the cluster. We subjectively determined the optimum number of clusters (six) by assessing the homogeneity of the PEA clines against the mean annual temperature gradient.

To investigate levels of standing variation, we calculated expected heterozygosity (*H*_e_) for each PEA in each population. The cluster‐mean *H*_e_ for each population is the mean *H*_e_ for each SNP within the cluster. We report standing variation as proportional polymorphism for each population: the proportion of SNPs within a cluster with *H*_e_ > 0.

## 3 RESULTS

### 3.1 Phenotypic clines

Population‐mean phenotypes for all four traits measured in the Vancouver seedling common garden exhibit moderate to strong clines relative to the temperature gradient of the study area (Figure 2 and Figure S14). Autumn cold injury and the timing of growth cessation show the strongest relationships with mean annual temperature. In general, trees from colder provenances initiated growth slightly earlier, ceased growth earlier, achieved less total growth, and exhibited less cold injury. Autumn cold injury in particular has a very strong relationship (*r* = .83) to mean annual temperature. Within‐population variation among individuals is generally uncorrelated among the four traits (Figure S3). However, within‐population variation of shoot mass is positively correlated with growth cessation day (*r* = .57) and weakly but significantly negatively correlated with growth initiation day (*r* = −0.18, *p* = 3E−12). This result may be due to the benign maritime climate of the common garden; seedlings with a longer growth period are not penalized by environmental constraints such as growing season frosts. Correlations of among‐population variation in growth cessation, fall cold injury, and shoot mass are moderate to strong. Growth initiation is poorly correlated with the other traits.

### 3.2 Phenotypic variation explained by geographic, climatic, and genomic data

**Figure 3:**
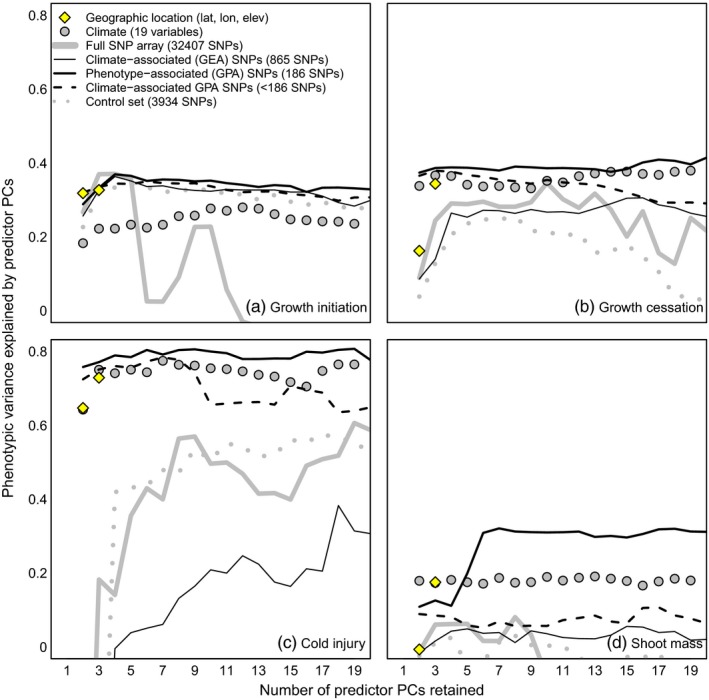
Seedling common garden phenotypic variance explained (*R* ^2^) for four traits by cumulative principal components of geography (diamonds), climate (circles), and several subsets of genomic data from a SNP array (lines). Each point is the cross‐validated *R* ^2^ of a multiple linear regression of population‐mean phenotype against the specified number of principal components of the predictor data. GEA SNPs (thin black line) are the pooled top 300 SNPs based on Bayes factor from each of the 19 climate variables. GPA SNPs (thick black line) are the top 1% of coding‐region SNPs (maximum of one SNP per contig) based on the *p*‐value of a population‐structure‐corrected linear association of allele frequencies to seedling phenotypes. Climate‐associated GPA SNPs (black dashed line) are GPA SNPs with a linear association with climate (see Section 2.4.1) and have *n* = 151, 144, 125, and 44 for the four traits, respectively. The control set is shown as a gray dashed line

The extent to which geographic, climatic, and genomic data explain phenotypic variation differs among traits (Figure 3). Differences in the explained phenotypic variation among traits generally exceed the differences among the three types of data (geographic, climatic, and genomic) within traits, consistent with the relative strengths of the phenotypic clines in Figure 2. Nevertheless, there are important differences in the relative explanatory content of geographic, climatic, and genomic data among traits. In general, geographic variables (yellow diamonds) are as predictive of seedling phenotypes as climatic variables (gray circles, Figure 3), consistent with local adaptation to geographically based climate in this species. The exception is growth initiation, where geographic variables are more explanatory than climate. The GPA SNPs (solid black line, Figure 3) are more explanatory than climate and geography in growth initiation and shoot mass but not growth cessation, where they are equivalent, and cold injury, where they are slightly inferior.

The relative explanatory power of different types of genomic data is consistent among traits (Figure 3) and provides several insights. GPA SNPs (solid black line) consistently have the highest explanatory power, as would be expected. Since the GPA SNPs are a subset of the full array (solid gray line), the difference between the GPA set and the full SNP array indicates the value of extracting the relevant genetic information from phenotypic associations. The climate‐associated GPA SNPs (black dashed line, Figure 3) explain less slightly less phenotypic variation than the full set of GPA SNPs, and substantially less for shoot mass. Climate‐associated GPA SNPs explain more phenotypic variation than climate variables for growth initiation, but less for shoot mass. The GEA SNPs identified using bayenv2 (Table S1) generally have low explanatory power to predict phenotypic variation, though for growth initiation, they have relatively high explanatory power equivalent to the GPA SNPs. There is a high overlap of GEA with GPA loci, with an average of 62.1% of GEA SNPs from various environmental variables found within 1,000 bp of GPA loci (range 0% for NFFD to 88% for EXT; *SD* = 21.2%), and a total of 44.5% of GPA SNPs found within 1,000 bp of the GEA loci found across environmental variables (Table S1). This is another line of evidence of the strong role of climate in driving phenotypic variation among populations. Figures S14–S16 show PCs 1–6 of allelic variation in the full SNP array, the control set, and the pooled GPA loci, respectively.

Both the control set and the full SNP array have explanatory relationships with phenotypes, as would be expected from population structure alone. However, these relationships are not as strong as those with geographic, climatic, and filtered genomic data (Figure 3). An equivalent analysis to Figure 3 using Random Forest regression instead of linear regression demonstrates that both the control set and full SNP array contain almost as much nonlinear explanatory information as the climatic and geographic variables (Figure S5). Further, some subsets of the control set exhibit linear relationships to phenotype that are as strong and stable as the relationships of GEA loci to phenotype (Figure S6).

Traits differ substantially in the dimensionality of their associated genomic information, that is, the number of PCs at which further gains in explanatory information are not achieved. Explainable variation in growth initiation, growth cessation, and autumn cold injury are predominantly described by the first two PCs (Figure 3). In contrast, six PCs are required to describe the explainable variation in shoot mass. The dimensionality of explanatory information in the different traits speaks to the complexity of genetic controls on the trait.

### 3.3 Climatic drivers of local adaptation

**Figure 4:**
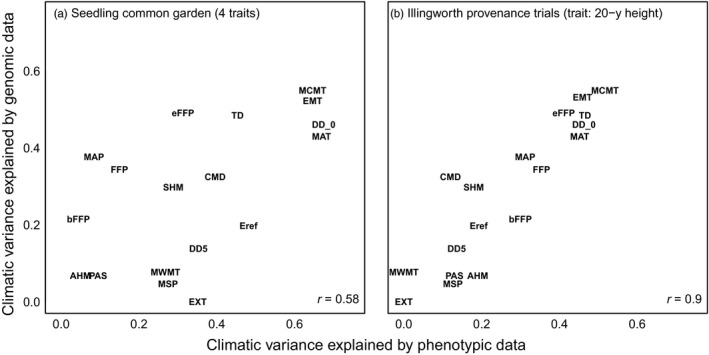
Climatic variable selection based on genomic versus phenotypic data in (a) the Vancouver seedling common garden and (b) the Illingworth provenance trials. Variance explained is the cross‐validated *R* ^2^ of a multiple linear regression of each climate variable (response variable) against the phenotypic or genomic predictor variable set. Genomic data (predictor variables for the y‐axis analyses) are four principal components of the minor allele frequencies for the top 300 GEA SNPs identified by bayenv2 for each climate variable. Phenotypic data (predictor variables for the x‐axis analyses) for panel A are population‐mean phenotypes for the four common garden traits presented in Figure 2. Phenotypic predictor data for panel B are 20‐year heights of the Illingworth lodgepole pine provenance trial. Climate variable acronyms are described in Table 1

Genotype–environment association loci and short‐term (3‐year) seedling common garden phenotypes have moderately similar relationships (*r* = .56) to the 19 climate variables (Figure 4a). This congruence is much stronger (*r* = .90) between the GEA loci and the longer‐term (20‐year) provenance trial (Figure 4b). Across both phenotypic datasets and the genomic GEA data, there is agreement that local adaptation is strongly associated with winter temperature variables: mean temperature of the coldest month (MCMT), degree‐days below 0°C (DD_0), winter–summer temperature contrast (TD), and extreme minimum temperature (EMT). Note that mean annual temperature can be considered primarily a winter variable in this study area because spatial variation in mean temperature along the latitudinal gradient of the study area is much stronger in winter than in other seasons. In the Vancouver common garden (Figure 4a), this congruence between genotypic and phenotypic relationships to climate variables is broken by summer temperature variables (Eref, EXT, DD5, and MWMT), which have moderate associations with phenotypes (x‐axis) but low associations with genotypes (y‐axis), and by mean annual precipitation (MAP), which is moderately associated with genomic but not phenotypic variation. The same pattern of these relationships is produced using either the full SNP array or the control SNPs in place of the GEA SNPs (Figures S6 and S7, respectively). The genomic data and seedling common garden phenotypes have covariation with the first five principal components of the 19 climate variables and show moderate agreement on the strength of this covariation (Figure S11).

### 3.4 Spatial scale of local adaptation to climate

**Figure 5:**
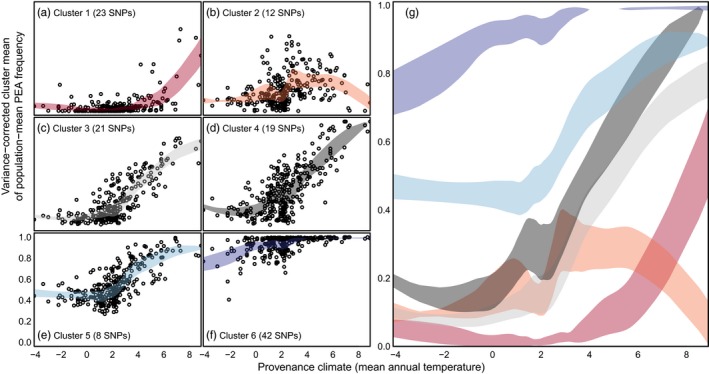
Genetic clines associated with autumn cold injury. (a–f) The 125 climate‐associated GWAS SNPs for autumn cold injury are clustered based on similarities in positive effect allele (PEA) frequencies across populations (*n* = 281). Each point is the mean of the PEA frequencies across clustered SNPs for one population, with a correction applied to restore the variance of the PEA frequencies following averaging. The colored bands in each plot, superimposed in panel g, are locally weighted 0.5‐standard deviation prediction intervals. Recall that the y‐axes are the frequency of PEAs that are associated with increased cold injury

All four seedling common garden traits exhibit linear phenotypic clines over many of the climatic gradients of the study area, where the strongest of these clines is autumn cold injury relative to mean annual temperature (Figure 2c; *r* = .83). To detect whether genetic clines for cold injury loci along environmental gradients are rangewide or localized (*sensu* Box 1), we examined the *n* = 125 locus subset of the 186 cold injury GPA candidates that are also moderately associated with the 19 climate variables (linear *R* ^2^ > 0.35; Figure S4). We clustered these 125 loci into six clusters based on their absolute PEA frequencies across populations (Figure S11). The population‐mean PEA frequencies of these six clusters have distinct clines (Figure 5) relative to the gradient in mean annual temperature across the study area (Figure 2). Clusters 2, 3, 4, and 5 show little clinal variation across provenance temperatures below 0°C, but have a clinal increase in PEA frequency across higher temperatures (Figure 5). Cluster 6 has essentially the opposite pattern, in that it shows clinal variation almost exclusively below 2°C mean annual temperature. The adaptive variation in cluster 6 is of interest, in the context of standing variation, because it is localized to a high degree relative to the other clusters. Cluster 1 has an inverse pattern to cluster 6 relative to provenance climate, and primarily reflects variation associated with the coastal ssp. *contorta*, which occur at MAT > 6°C. Cluster 2 exhibits increased variation in the interior of BC, which is reversed in the warmer climates of the coast. Results equivalent to Figure 5 for the other three traits are provided in Figures S18–S20.

**Figure 6:**
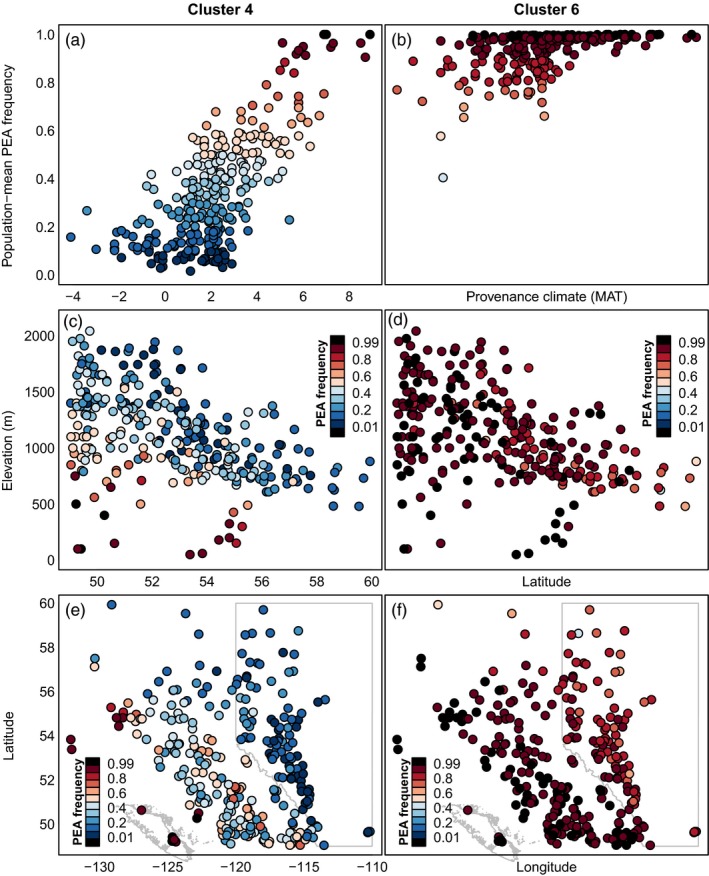
Contrasting geographic patterns of standing variation in rangewide and localized genetic clines associated with autumn cold injury. A rangewide cline (cluster 4, left column) and a localized cline (cluster 6, right column) relative to the mean annual temperature gradient (MAT) in the sampled populations (a and b, respectively) as previously shown in Figure 4d,f. These clines are also compared across latitude and elevation (c, d), and latitude and longitude (e, f). Populations are colored with respect to PEA frequency (alleles that are associated with an increase in autumn cold injury)

To contrast the extent of rangewide versus localized clines, the geographic patterns of allele frequencies in clusters 4 and 6 are shown in Figure 6. Cluster 4 represents the dominant rangewide genetic cline over the study area and is largely parallel with clusters 3 and 5. Cluster 6 is the complementary cline to cluster 4 as it reflects adaptive variation for cold hardiness in boreal but not temperate populations. Cluster 4 has a strong cline with respect to the joint thermal gradient of latitude and elevation (Figure 6c). Putatively adaptive alleles of cluster 6 are predominantly found in the Boreal climates of Northern Alberta, Northeastern BC, and the eastern foothills of the Rocky Mountains (Figure 6f). Unlike cluster 4, cluster 6 does not have a pronounced elevational cline at low latitudes (Figure 6d). Except for two coastal populations, all populations have standing variation in some of the adaptive alleles in each cluster, though several populations west of the Rocky Mountains (i.e., in British Columbia) have no standing variation in at least half of the cluster 6 loci (Figure S12).

## 4 DISCUSSION

This study uses a large sample of *P. contorta* populations from across western Canada to evaluate genomic data, relative to phenotypic and climatic data, as a basis for assisted gene flow and genetic conservation. In Introduction, we posed three research questions related to this objective. The first was: *what is the relative value of genomic data versus climatic and geographic data in explaining variation in locally adapted traits?* For all traits, the control and GEA SNPs explained far less variation than climate, GPA SNPs, or even geographic coordinates (Figure 3). This suggests that genomic data are most useful as a complement to, rather than a replacement for, phenotypic and climatic data as guidance for assisted migration. The second question was: *can genomic data identify the climatic drivers of local adaptation?* Genotype–environment associations had strong agreement with both a long‐term provenance trial and a short‐term seedling common garden on the climatic drivers of local adaptation, namely winter temperature‐related variables (Figure 4). This suggests that genomic data can be a viable option for identifying the key climatic controls on productivity and lifetime fitness and may even be more reliable for this purpose than seedling traits in some contexts (Figure S9). The third question was: *what is the spatial scale of local adaptation in climatically adaptive traits?* We did not find compelling evidence for highly localized genetic clines at scales that would constrain local seed transfer to scales finer than those indicated by phenotypic data or necessitate geographically small genetic conservation units (Figures 5 and 6).

### 4.1 Phenotypic variation explained by geographic, climatic, and genomic data

The predictive power of climate variables, geography, and genotypes varied greatly among seedling traits. It is widely recognized that cold hardiness shows strong population differentiation in most temperate and boreal tree species (Aitken & Bemmels, 2016; Alberto et al., 2013; Howe et al., 2003), and we found strong population differentiation for cold injury, as well as high predictability of cold injury from climatic, geographic, and GPA SNP data (*R* ^2^ > 0.6). However, the remaining traits were not as strongly predicted with any of the given data (*R* ^2^ < 0.5; Figure 3). Variability in the predictive ability among traits for a given data source or among data sources for a given trait may be due to several factors (discussed in Lind et al., 2018): (a) how well each phenotype is correlated with lifetime fitness; (b) the degree to which the trait is polygenic; (c) the mode of gene action underlying the genetic architecture of the trait (e.g., additive, epistatic/GxE, or pleiotropic); (d) the primary source of genetic variation in a trait (i.e., protein‐coding or regulatory regions); (e) the degree to which selection has structured variation within the species (i.e., the joint effects of selective forces and demographic dynamics); or (f) shortcomings of methodologies (e.g., correcting for population structure that could remove adaptive signals that covary with demography).

While this study focused on relatively few seedling traits, there are undoubtedly many other traits at various life history stages that have population differences associated with local climate (e.g., biotic and abiotic responses, reproduction, and tree form). Our GPAs specifically identify SNPs associated with our focal seedling traits, and so it is not surprising that the GPA SNPs from individual seedling traits were better predictors of a given trait than the GEA SNPs (Figure 3). Even so, the GPA SNPs were consistently the best set of markers for explaining variation in phenotypes (Figure 3), emphasizing the added value of these candidate loci. Climate also consistently explained phenotypic variation well, relative to genomic data, for traits other than growth initiation. Geographic coordinates (latitude, longitude, and elevation) predicted all seedling traits quite well, reflecting the success found in the vast body of older genecological literature in forest trees that used geographic variables as a proxy for climate before spatial climatic data became widely available.

In line with expectations of polygenic architectures for most of the traits (i.e., causative sites throughout the genome), the entire SNP array (~31K SNPs) was able to predict some of the variation in these traits. Control SNPs selected randomly from noncoding regions of the genome were also able to explain a substantial portion of phenotypic variation in all traits except shoot mass (Figure 3) and were equivalent to all other data sources as a predictor set for Random Forest regressions (Figure S5). The predictive power of control SNPs emphasizes the potential to confound neutral population structure with adaptive variation or to overcorrect for population structure, and, as a result, overlook adaptive markers, particularly for species whose demographic history is aligned with environmental gradients. In this case, the postglacial expansion of lodgepole pine likely matches the strong latitudinal gradient of winter temperatures. Since the analyses identifying GEA and GPA SNPs both adjusted for population structure, we may have eliminated some loci involved in local adaptation from consideration through this adjustment. Even so, given the choice of markers used to correct for structure, our hits likely represent a conservative approach. Combined with the signal from the two common garden experiments (Figure 4), as well as the overlap of loci between GEA and GPA despite a large marker set used for testing (Table S1), our results suggest strong influence from winter‐related variables driving adaptive genetic variation.

### 4.2 Climatic drivers of local adaptation

To design an assisted gene flow strategy that matches populations with suitable sites based on current and near‐future climates, it is important to understand the climatic factors that have driven local adaptation. Once the key climatic factors for local adaptation are identified, a climate distance metric can be constructed to match seed sources with sites (e.g., Climate‐Based Seed Transfer, O'Neill et al., 2017, and Seedlot Selection Tool, https://seedlotselectiontool.org/sst/). Our GEA results for individual climate variables ranked the variable importance similar to those identified based on growth in a 20‐year field provenance trial and, to a lesser extent, to our seedling common garden phenotypes. Both sets of phenotypic data identified winter temperature variables including mean coldest month temperature, degree‐days below 0°, and extreme minimum temperature as important drivers of local adaptation. Other studies of these provenances (e.g., Liepe et al., 2016) and other populations of lodgepole pine in western Canada (e.g., McLane, Daniels, & Aitken, 2011; Rweyongeza, Dhir, Barnhardt, Hansen, & Yang, 2007; Wang et al., 2010) corroborate these climatic variables as strong historic drivers of adaptation and differentiation, and at relatively broad spatial scales (Liepe et al., 2016). Nevertheless, the result that our set of control markers produced nearly equivalent climate variable rankings to the GEA set (Figure 4 vs. Figure S8) indicates that the substitution of genomic for phenotypic data needs to be approached with some caution.

Future pressures from drought are expected to become increasingly relevant for lodgepole pine populations as climate change progresses over the next century (McLane et al., 2011; Monserud, Huang, & Yang, 2006; Monserud, Yang, Huang, & Tchebakova, 2008). GEA–climate relationships were stronger than field phenotype–climate relationships for summer precipitation‐related variables such as mean summer precipitation and cumulative moisture deficit (Figure 4). This suggests that water availability might result in diversifying selection across populations. A previous study with these populations found no significant population variation for drought‐related seedling traits including stable carbon isotope ratios and biomass allocation to roots (Liepe et al., 2016). However, it did not include populations from drier provenances in the southern portion of the species range, and these may show stronger drought adaptation. Drought hardiness is also difficult to phenotype as there are many potential component traits involved in tolerance or avoidance.

None of the phenotypes we analyzed represent lifetime fitness. Nonetheless, the concordance of climatic drivers of seedling phenotypes, 20‐year growth in the field, and genomic data are encouraging (Figure 4). While it is not feasible to assess lifetime fitness in long‐lived forest trees or to determine all of the component traits affecting fitness (Alberto et al., 2013), seedling shoot mass is likely one of these component traits. Trees that achieve larger sizes within the available frost‐free period for growth will generally have higher fecundity as they have larger crowns with more sites for pollen and seed cone production (Aitken & Bemmels, 2016). Forest managers are also ultimately interested in tree size for wood production or carbon sequestration, and trees with good juvenile growth are likely to grow well in a restoration context. In our study, the ability of climatic and genomic data to describe among‐population phenotypic variation was lower for shoot mass than for the other seedling traits. Tree size is the product of many other component traits affecting seedling health and vigor, including phenology (which we analyzed directly as growth initiation and cessation), abiotic stress tolerance (including cold injury), resistance to insects and diseases, resource acquisition and allocation, physiological processes, and cell density. It is likely that loci underlying variation in growth have pleiotropic effects and that they respond to selection through trade‐offs in the various fitness consequences of component traits contributing to growth.

Which of these data sources—seedling phenotypes, field phenotypes, or genotypes—should be considered the standard against which the others are compared? One could argue that field‐based growth over two decades better reflects meaningful population differences expressed in typical habitat. On the other hand, the precision phenotyping of seedlings for phenology and cold hardiness is difficult or impossible in long‐term field trials, and these traits should be strongly linked with climate for boreal, sub‐boreal, and montane species where they are critical to fitness. Finally, it may be that the GEA–climate patterns provide the best indication of long‐term selection as they may reflect periodic, episodic extreme climatic events causing injury and mortality that are not observed even over long field experiments. In any event, given the extensive overlap in top climate variables among these methods, we suggest that GEA approaches can rapidly provide information on climatic drivers of local adaptation for the design of assisted gene flow strategies when phenotypic data are not available. However, the potential for population structure to confound GEA approaches and the poor performance of GEA loci in predicting locally adapted seedling traits both suggest caution is warranted.

### 4.3 Spatial scale of local adaptation to climate

We evaluated variation at adaptive loci against a model of localized versus rangewide genetic clines (Figure 1; sensu Barton, 1999) along climatic temperature gradients (Figure 2). We found evidence of both localized and broad‐scale genetic clines for clusters of SNPs associated with autumn cold injury (Figures 5 and S4). Overall, the genetic clines associated with autumn cold injury do not exhibit the strongly sequential, localized clines envisioned by Barton (1999) and Savolainen et al. (2007), nor are all genetic clines strictly coincident across the range of environments, but rather fit a model intermediate to the hypothetical scenarios illustrated in Figure 1b,c. Our study sampled provenances over only half of the species' latitudinal range. It may be that sequential localized genetic clines would be more evident if our study included the full species range. While some clines for the major adaptive clusters we identified are largely variable across the range, there is a group of 42 SNPs that all show clines in the boreal region of the study area, but not in warmer areas (cluster 6 in Figure 5). These clines complement those of several other clusters for SNPs that are relatively invariant in the boreal portion of the range but vary in warmer regions (clusters 1, 3, and 4 in Figure 5). For instance, cluster 6 alleles conferring cold hardiness (the alternate PEA allele) have reduced standing variation in warmer provenances west of the Rocky Mountains and follow both elevational and latitudinal patterns of temperature clines (Figures 5 and 6). Failure to detect polymorphisms for these SNPs in these populations may be an artifact of small sample sizes (6 < *n* < 13) in most of the studied populations (Figure S2). Nevertheless, these results indicate low genetic diversity in the boreal‐associated alleles of cluster 6 in these locations. The absence of these alleles may be a limiting factor in seed transfer from sub‐boreal to boreal climates, or across the Rocky Mountains. This localization may be indicative of alleles conferring additional cold hardiness in the coldest areas of the sampled range that may have trade‐offs in the warmer areas (e.g., via pleiotropy or GxE such as conditional neutrality). Even so, the alleles in cluster 6 were not associated with the other phenotypes in our study (while all other clusters had associations to at least three phenotypes). Future investigation is warranted, as the lack of pleiotropy inferred from associations to multiple phenotypes in cluster 6 may be a function of the cluster's sample size, of linkage to unsampled antagonistic (regulatory) sites, conditional neutrality underlying gene action (or other GxE), of unmeasured phenotypes important to adaptation, or of other statistical and methodological shortcomings.

While our results suggest that localized genetic clines (Figure 5), and populations associated with low genetic diversity in adaptive alleles (Figures 6 and S8), are evident in lodgepole pine, we did not find compelling evidence for localized genetic clines at scales that would constrain local seed transfer more narrowly than previous estimates of adaptive scales based on phenotypes (*cf*. Figure 4 in Liepe et al., 2016; Wang et al., 2010; Ukrainetz, Yanchuk, & Mansfield, 2018) or current seed transfer policy would suggest (O'Neill et al., 2017; Ying & Yanchuk, 2006), nor at scales that would necessitate highly localized spatial genetic conservation units. At present, British Columbia's genetic conservation program for forest trees uses British Columbia's 16 Biogeoclimatic Ecological Classification (BEC) zones to assess adequacy of both in situ (Hamann, Aitken, & Yanchuk, 2004; Chourmouzis, Yanchuk, Hamann, Smets, & Aitken, 2019) and ex situ (Krakowski et al., 2009) genetic conservations for all 50 of BC's native tree species. If other species show patterns of distribution of adaptive diversity similar to lodgepole pine, continued management of conservation populations within these ecological zones should be sufficient (Liepe et al., 2016).

### 4.4 Conclusions

Historically, the spatial scales over which local adaptation occurs have been inferred from both short‐ and long‐term transplant experiments. Only recently has the technology been available to study the spatial distribution of adaptive variation at loci across the genome. This new source of insight into local adaptation comes at a time when climate change creates an imperative for mitigating inevitable risks of productivity loss and threats to natural populations across forestry, agricultural, and natural systems. The common sources of data used toward such purposes, such as field provenance trials, seedling common gardens, scale‐free spatial climatic data, and genomic studies, however, come with varied logistical limitations and are not always feasible or appropriate in every situation. The large number of phenotyped and genotyped populations in this study allows us to quantify and compare detailed spatial and climatic patterns of adaptive variation, and to assess their utility for planning assisted gene flow, the need for in situ and ex situ genetic conservation, and the potential for populations to adapt to new climates without intervention. We found that climate, geography, and SNPs associated with climate‐related seedling phenotypes within populations were good predictors of variation among populations and could all play an effective role in designing assisted gene flow strategies. SNPs associated with climate alone were not good predictors of these seedling traits; however, they did identify the same primary climatic drivers of adaptation as common garden experiments and so could inform variable selection for estimating climatic transfer distances for assisted gene flow. Our analysis of genetic clines identified a set of alleles exclusive to boreal populations that are associated with seedling cold hardiness, demonstrating the utility of genomic analysis in identifying potential constraints to seed transfer. While our data are for lodgepole pine, we hope these results will inform and accelerate climate adaptation efforts with other widespread species.

## CONFLICT OF INTEREST

None declared.

## AUTHOR CONTRIBUTIONS

SNA and CRM conceived this project. IRM performed Vancouver seedling common garden experiments and phenotyping. JBY and IRM compiled SNP tables. CRM designed the analyses with input from IRM, SNA, and BML. IRM performed GPA analysis. BML performed GEA analysis. CRM contributed to figures. TW analyzed Illingworth trial. SNA, BML, CRM, and JBY contributed to the introduction. IRM and CRM contributed to the methods. CRM contributed to the results. CRM, SNA, BML, and JBY contributed to the discussion. CRM contributed to the supporting information document.

## Data Availability

All raw data supporting this publication are archived at https://doi.org/10.5061/dryad.56j8vq8 (MacLachlan, 2019).
