# Supplementary material for: Evaluating genomic data for management of local adaptation in a changing climate: A lodgepole pine case study
Source: Evol Appl. 2019 Sep 30;13(1):116–31. doi: 10.1111/eva.12871 (PMC6935591; doi:10.1111/eva.12871)
Supplement: Supplementary file 1 [file EVA-13-116-s001.pdf]

Evaluating genomic data for management of local adaptation in a changing climate: A lodgepole pine case study

Supplementary Information

Colin R. Mahony[1*], Ian R. MacLachlan[1], Brandon M. Lind[1],
Jeremy B. Yoder[1,2], Tongli Wang[1], and Sally N. Aitken[1]

[1]Centre for Forest Conservation Genetics and Department of Forest and Conservation Sciences, University of British Columbia, Canada
[2]Department of Biology, California State University Northridge, USA
*Corresponding Author: c_mahony@alumni.ubc.ca

August 18, 2019

Table S1. **Overlap between genotype-environment associations (GEA) from `bayenv2` and genotype-phenotype associations (GPA).** The GEAs column indicates the number of environmentally associated loci that met our filtering criteria (locus in top 300 ranks for Bayes factor across all three chains, and also in top 300 ranks for *rho* across all three chains, after filtering for one SNP per contig). Numbers in phenotypic columns indicate the overlap of GEAs with GPA hits (i.e., the same position in each association). Unique GPA Hits is the unique number of loci across these phenotypic columns for a given environmental variable. The last row indicates the unique number of SNPs from a given column. Environmental abbreviations as in Table 1 of main text.

Environmental Variable	GEAs	Budbreak	Budset	Cold injury	Shootmass	Unique GPA Hits
AHM	206	40 (18)	74 (31)	10 (7)	1 (1)	79 (36)
CMD	228	58 (24)	95 (41)	35 (17)	24 (7)	102 (46)
DD5	172	53 (19)	35 (8)	55 (17)	51 (5)	88 (26)
DD_0	175	58 (30)	67 (34)	52 (21)	36 (10)	95 (50)
EMT	141	54 (22)	29 (12)	51 (19)	33 (10)	76 (32)
EXT	25	11 (3)	17 (3)	18 (3)	17 (3)	19 (3)
Eref	223	65 (18)	98 (41)	43 (15)	35 (7)	109 (47)
FFP	194	63 (17)	31 (8)	58 (15)	44 (9)	100 (24)
MAP	135	40 (27)	53 (27)	13 (7)	0 (1)	65 (39)
MAT	213	85 (39)	67 (32)	61 (23)	41 (10)	121 (56)
MCMT	177	53 (22)	30 (11)	59 (20)	34 (8)	82 (32)
MSP	203	46 (24)	78 (43)	21 (16)	11 (9)	83 (48)
MWMT	135	21 (8)	35 (12)	38 (15)	47 (8)	56 (16)
NFFD	1	0 (0)	0 (0)	0 (0)	0 (0)	0 (0)
PAS	100	0 (2)	3 (5)	9 (2)	3 (1)	15 (8)
SHM	224	53 (24)	88 (42)	32 (17)	22 (8)	97 (47)
TD	164	14 (10)	11 (12)	35 (15)	11 (8)	38 (21)
bFFP	162	55 (19)	27 (8)	54 (16)	42 (9)	89 (26)
eFFP	167	62 (17)	31 (8)	59 (14)	44 (10)	100 (25)
unique loci	865	129 (69)	132 (74)	112 (54)	59 (22)	256 (129)

Table S2. Pearson's correlation between the 19 climate variables used in this study.

	MAT	MWMT	MCMT	TD	MAP	MSP	AHM	SHM	DD_0	DD5	NFFD	bFFP	eFFP	FFP	PAS	EMT	EXT	Eref
MWMT	0.6																	
MCMT	0.9	0.2																
TD	-0.6	0.2	-0.9															
MAP	0.3	-0.1	0.5	-0.6														
MSP	-0.3	-0.2	-0.2	0.1	0.2													
AHM	0.3	0.5	0.0	0.2	-0.7	-0.4												
SHM	0.5	0.5	0.3	-0.1	-0.2	-0.9	0.6											
DD_0	-0.9	-0.3	-1.0	0.9	-0.4	0.2	-0.1	-0.4										
DD5	0.8	1.0	0.4	0.0	0.0	-0.2	0.5	0.6	-0.5									
NFFD	0.8	0.7	0.6	-0.4	0.5	-0.1	0.1	0.4	-0.6	0.8								
bFFP	-0.6	-0.8	-0.3	0.0	-0.3	-0.1	-0.1	-0.3	0.3	-0.8	-0.9							
eFFP	0.8	0.5	0.7	-0.5	0.6	-0.1	-0.1	0.3	-0.7	0.7	1.0	-0.8						
FFP	0.7	0.7	0.5	-0.2	0.5	0.0	0.0	0.3	-0.5	0.8	1.0	-1.0	0.9					
PAS	-0.1	-0.5	0.2	-0.4	0.6	0.0	-0.7	-0.2	-0.1	-0.5	-0.2	0.4	0.0	-0.2				
EMT	0.9	0.4	0.9	-0.7	0.6	-0.3	-0.1	0.4	-0.8	0.6	0.9	-0.6	0.9	0.8	0.1			
EXT	0.6	0.9	0.2	0.1	-0.2	-0.5	0.6	0.7	-0.3	0.9	0.5	-0.5	0.4	0.5	-0.4	0.4		
Eref	0.8	0.8	0.5	-0.2	-0.1	-0.4	0.6	0.7	-0.6	0.8	0.5	-0.5	0.4	0.5	-0.4	0.5	0.9	
CMD	0.5	0.5	0.3	-0.1	-0.3	-0.9	0.7	1.0	-0.4	0.5	0.3	-0.2	0.2	0.2	-0.2	0.4	0.8	0.7

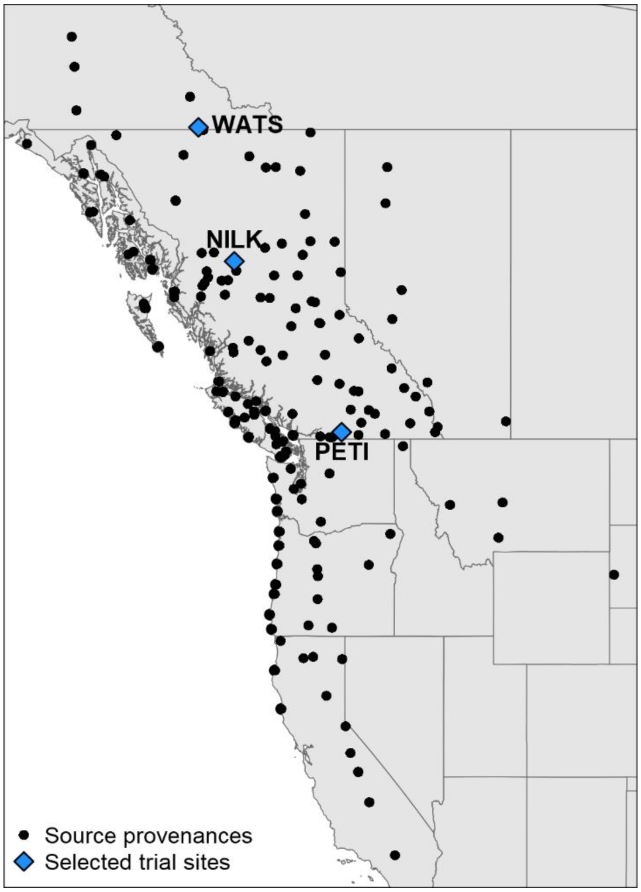

Figure S1: Source provenances and selected trial sites for the Illingworth lodgepole pine provenance trial.

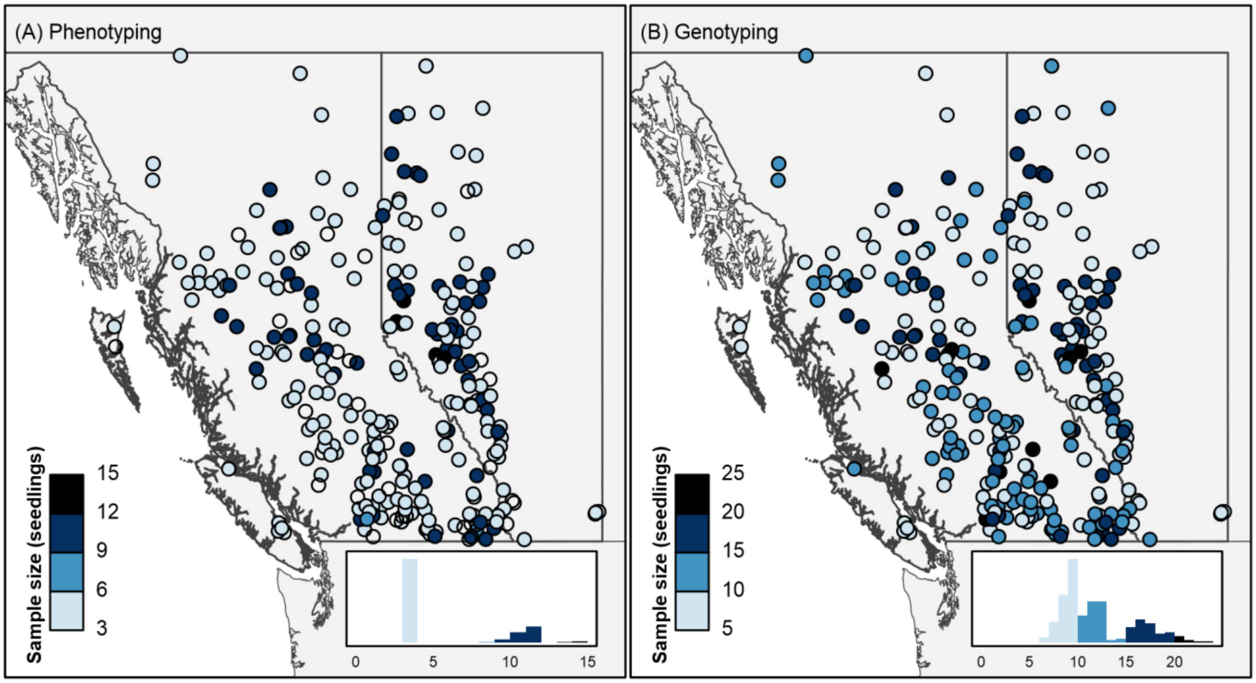

Figure S2: Sample size of common garden phenotyping and genotyping. SNP array genotyping was conducted on phenotyped common garden seedlings and an additional sample of seedlings grown in a growth chamber.

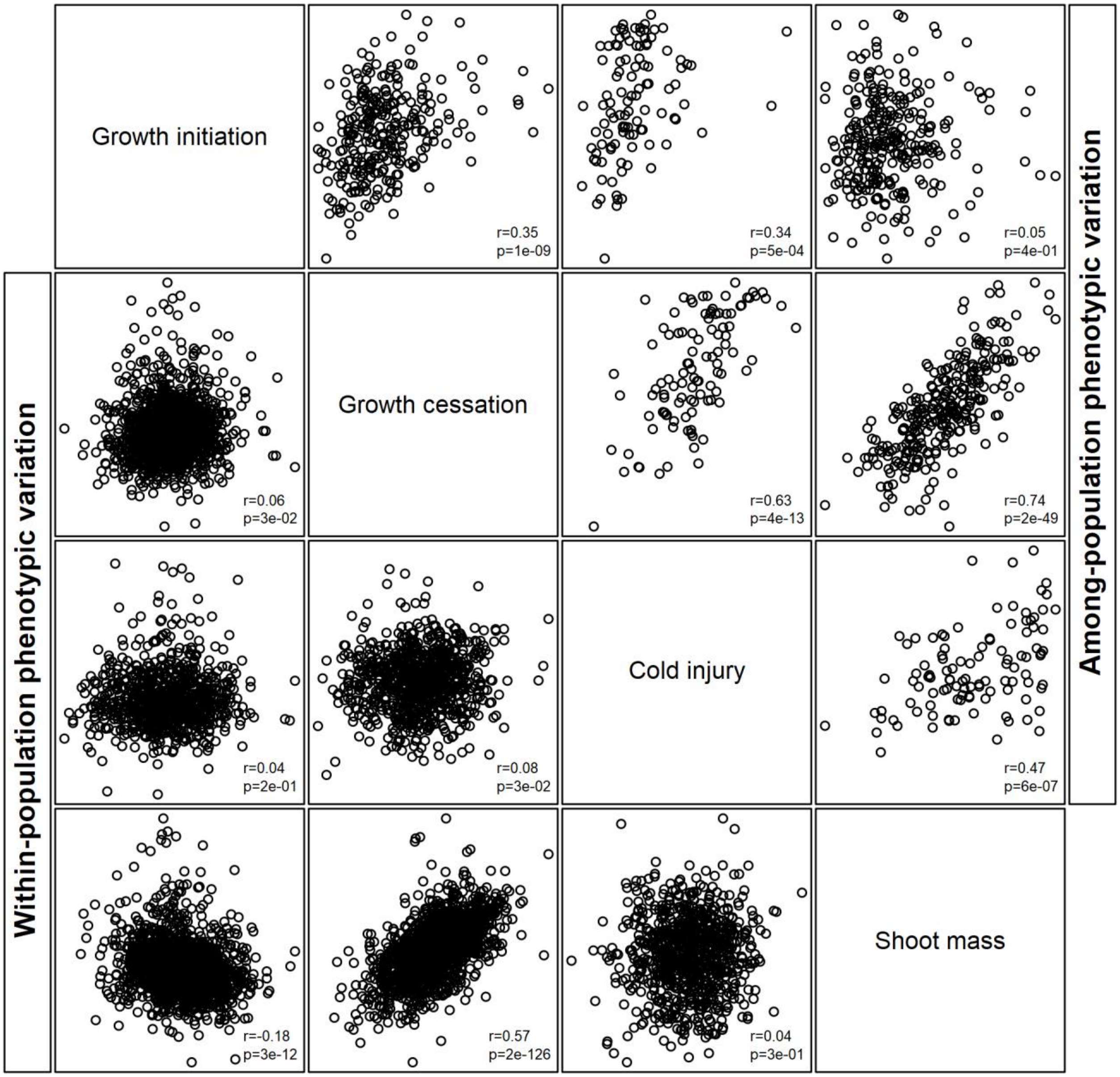

Figure S3: Among- and within-population relationships between the four traits. Among-population variation is the variation of population-mean phenotypic values. Within- population variation is the variation of individual seedling phenotypes that have had their population-mean phenotypic value subtracted.

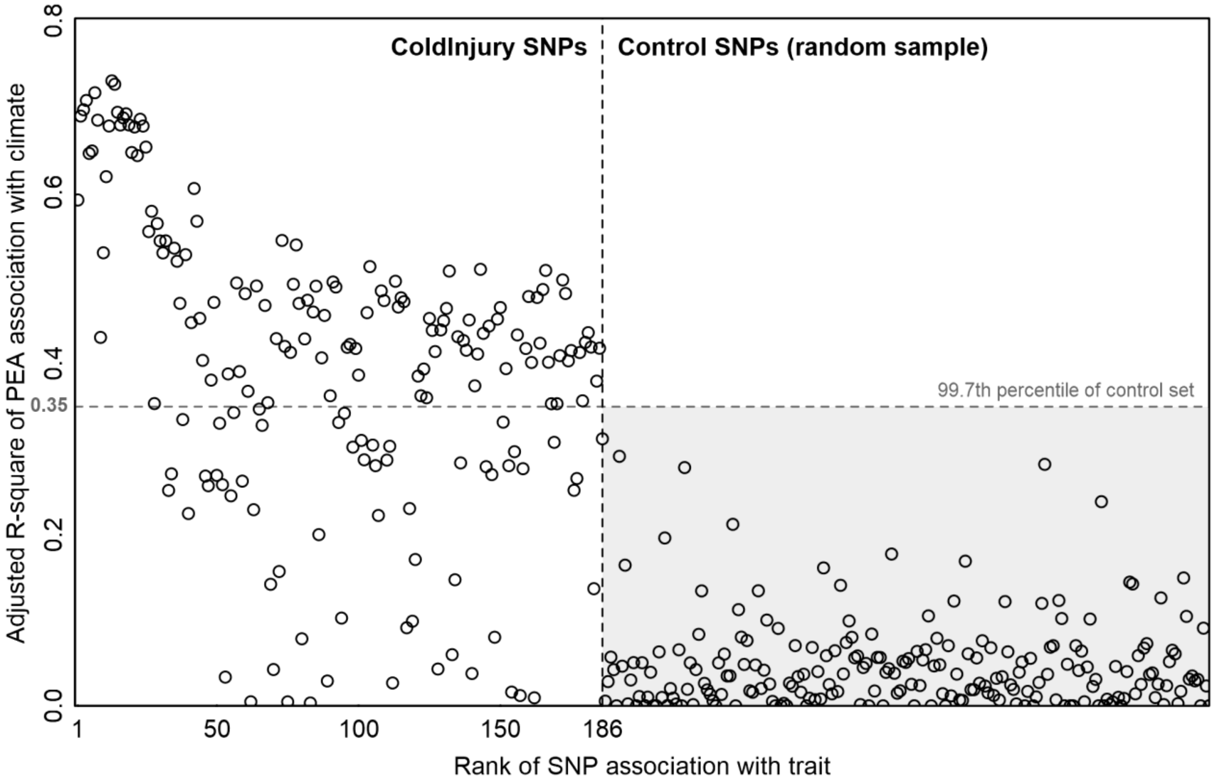

Figure S4: Relationship of GPA-selected loci to climate. The y axis is the adjusted $\boldsymbol{R^2}$ of a multiple regression of population-mean PEA frequency (response variable) to the five principal components of the 19 bioclimate predictor variables. PEAs are arranged in order of increasing GPA $\boldsymbol{p}$-value (decreasing significance), with a random sample of control SNPs shown for comparison. The 99.7th percentile of the association to climate in the control set is used as a threshold for selecting climate-associated PEAs.

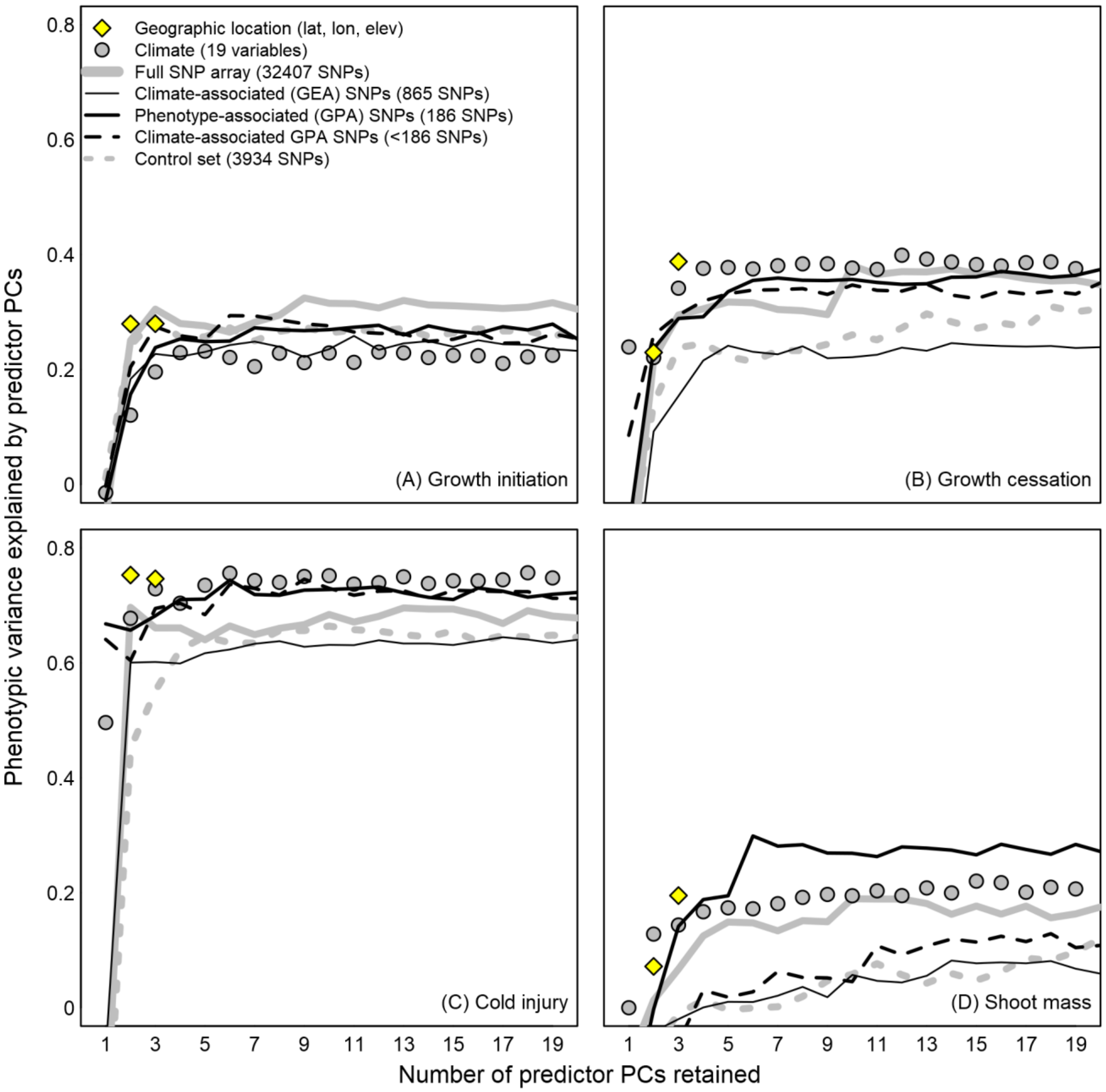

Figure S5: Equivalent analysis for Figure 3, using Random Forest regression instead of linear regression. Each point is the pseudo-R^2 of a Random Forest regression of population-mean phenotype against the specified number of principal components of the predictor data. GEA SNPs (thin black line) are the pooled top-300 SNPs based on Bayes factor from each of the 19 climate variables. GPA SNPs (thick black line) are the top 1% of coding-region SNPs (maximum of one SNP per contig) based on the p-value of a population-structure-corrected linear association of allele frequencies to seedling phenotypes. Climate-associated GPA SNPs (black dashed line) are GPA SNPs with further support for strong association to climate (see methods). The control set is shown as a grey dashed line

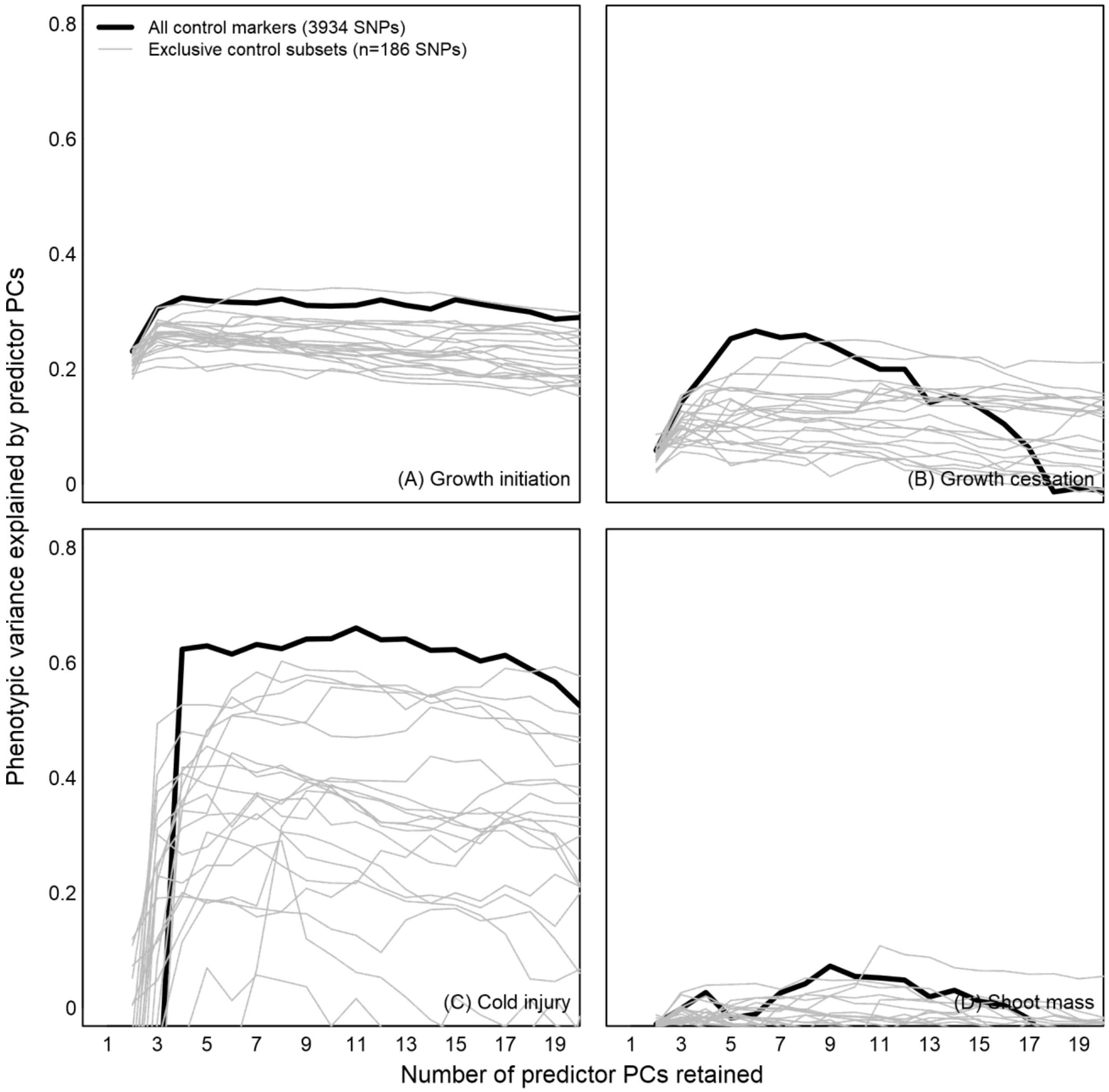

Figure S6: Explanatory power of small subsets of control SNPs. Each point is the cross-validated $\boldsymbol{R^2}$ of a multiple linear regression of population-mean phenotype against the specified number of principal components of minor allele frequency in an n=186 subset of control SNPs. Each grey line is a different subset, selected sequentially from the control set. The black line is the equivalent analysis for the full control set.

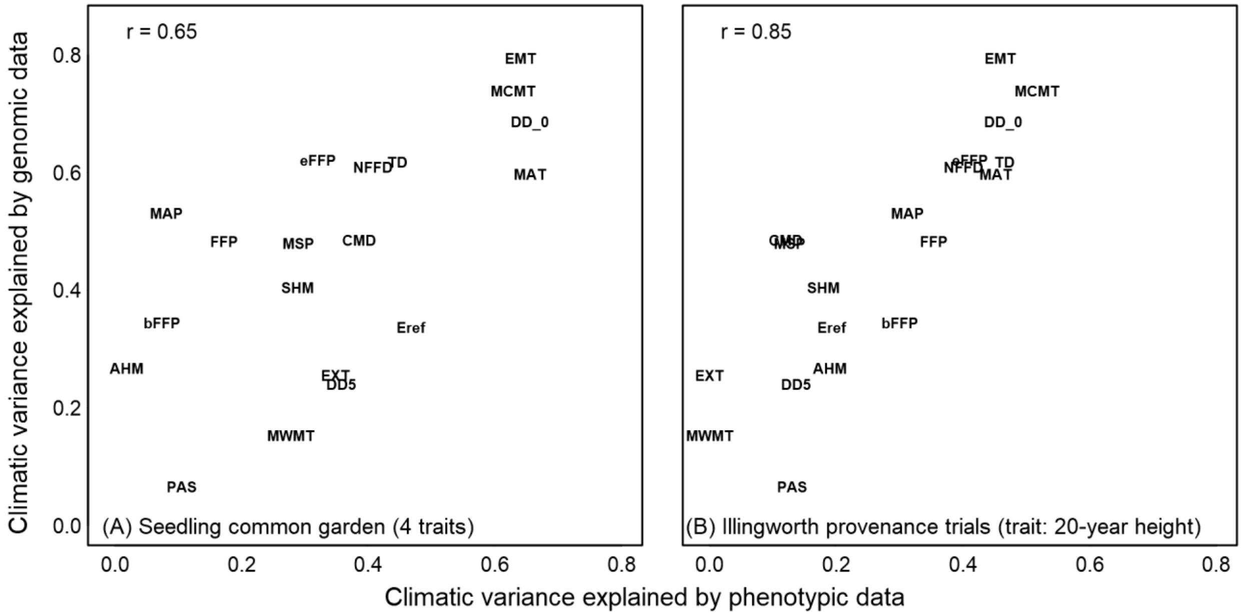

Figure S7: Climatic variable selection based on phenotypic vs genomic data, equivalent to Figure 4 except using the full SNP array instead of GEA SNPs. Variance explained is the cross-validated $\boldsymbol{R^2}$ of a multiple linear regression of each climate variable (response variable) against the phenotypic or genomic predictor variable set. Genomic data (predictor variables for the y-axis analyses) are four principal components of the minor allele frequencies for the full SNP array (n=31634 SNPs). Phenotypic data (predictor variables for the x-axis analyses) for panel A are population-mean phenotypes for the four common-garden traits presented in Figure 2. Phenotypic predictor data for panel B are 20-year heights of the Illingworth lodgepole pine provenance trial. Climate variable acronyms are described in Table 1.

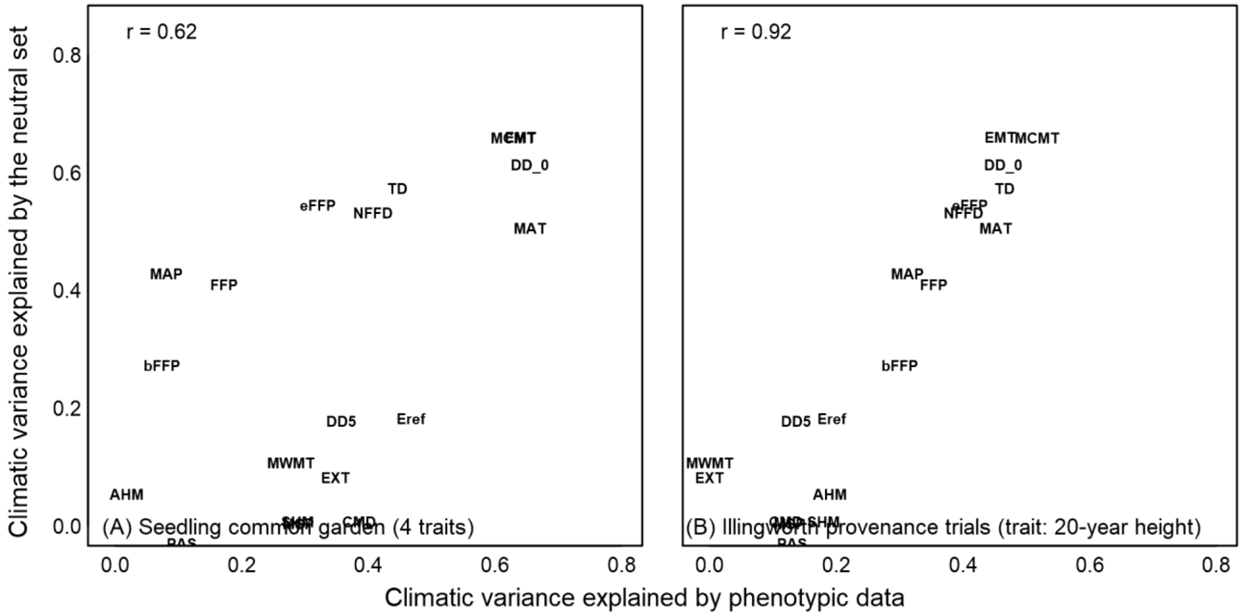

Figure S8: As in Figure S7 above, but using the control set (n=3934 SNPs) instead of the full SNP array.

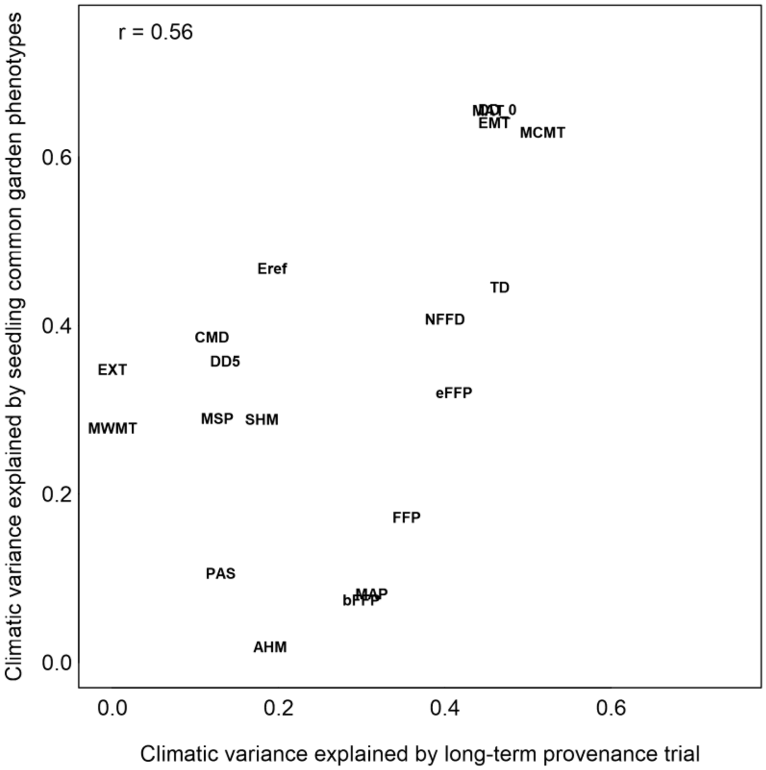

Figure S9: Climatic variable selection based on seedling common garden phenotypes vs. long-term provenance trial heights. Variance explained is the cross-validated $\boldsymbol{R^2}$ of a multiple linear regression of each climate variable (response variable) against the phenotypic predictor variable set. Phenotypic predictor data for the x-axis are 20-year heights of the Illingworth lodgepole pine provenance trial. Predictor variables for the y-axis are population-mean phenotypes for the four common-garden traits presented in Figure 2. Climate variable acronyms are described in Table 1.

71

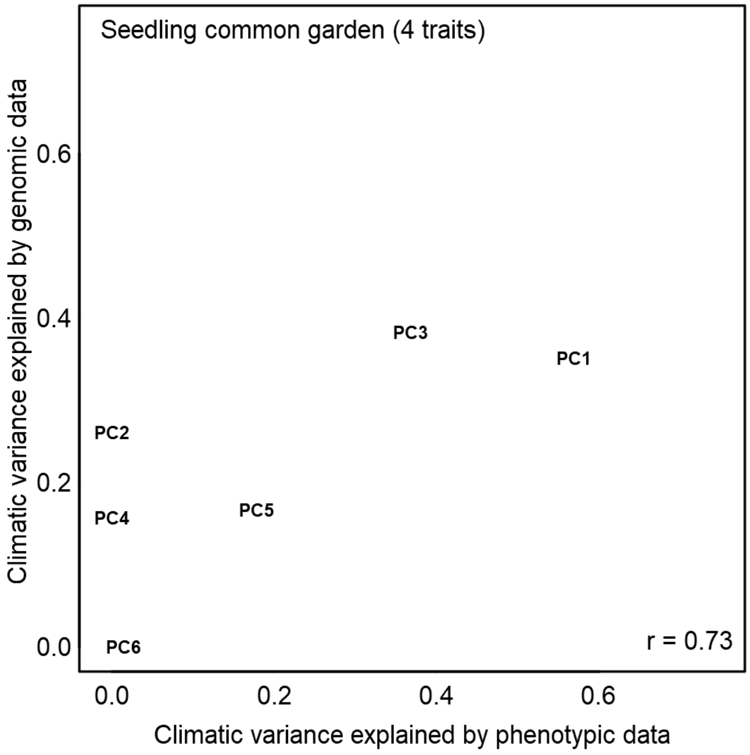

Figure S10: Equivalent to Figure 4A, but substituting the raw climate variables with the principal components of among-population variation in the 19 climate variables. PCs7-19 are not shown, as they have R^2=0 to both phenotypic and genomic data.

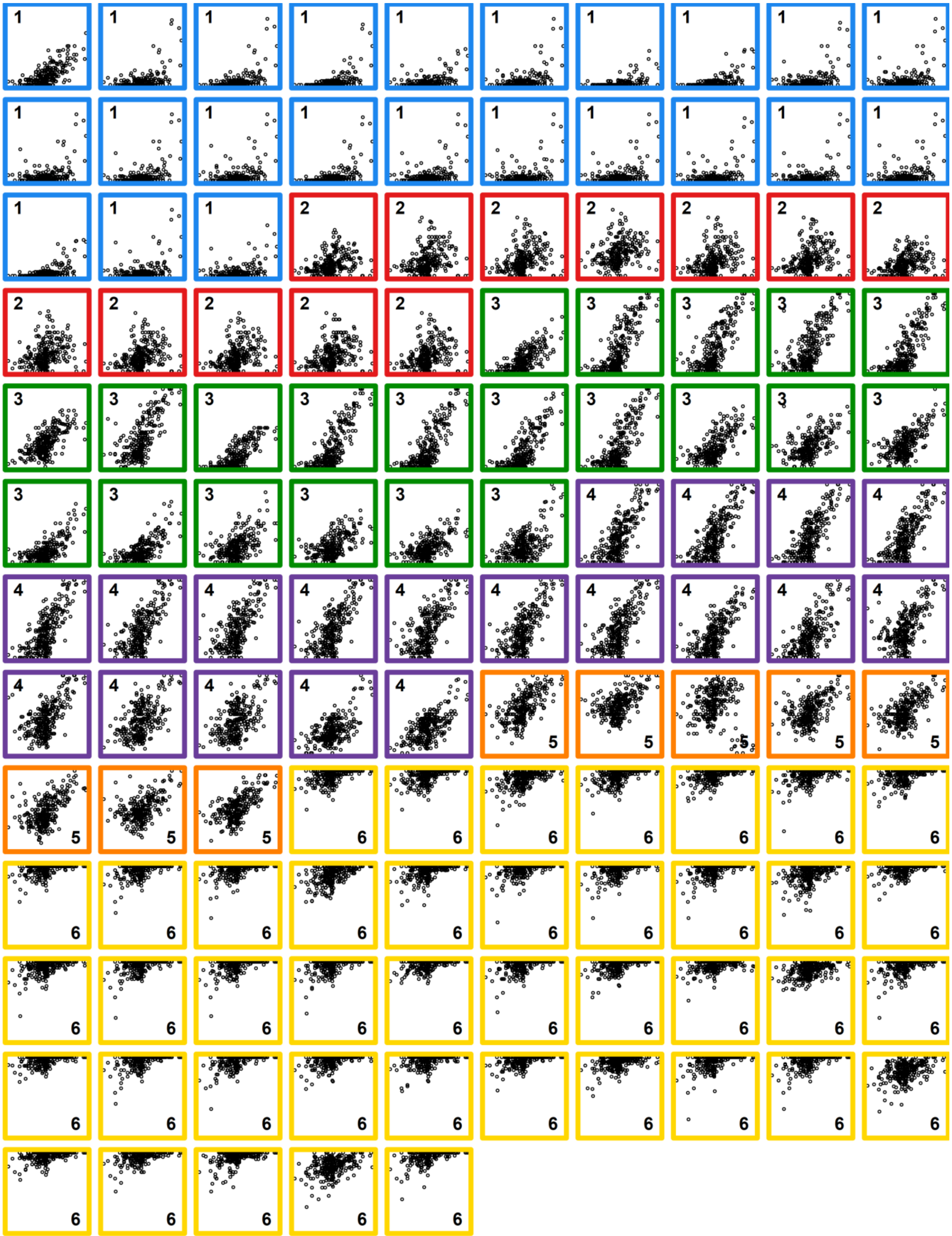

Figure S11: Genetic clines of climate-associated (R^2 > 0.35) GPA loci for autumn cold injury. Loci are clustered by PEA frequency across provenances. The x axis is mean annual temperature; the y axis is population-mean PEA frequency.

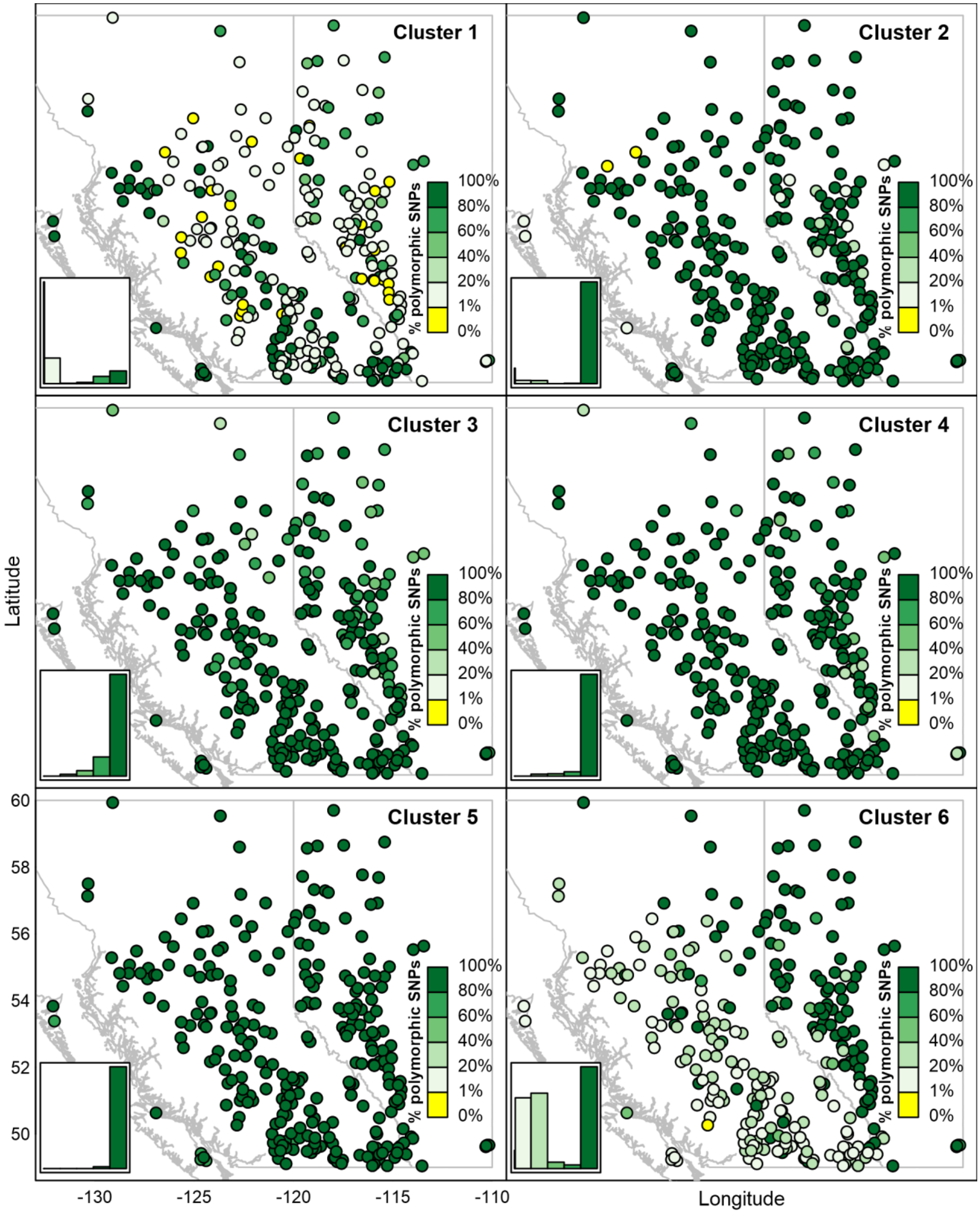

76
77 **Figure S12:** proportional polymorphism, by cluster, for each population: The percentage of the SNPs in each cluster
78 that have standing variation in both alleles, i.e., $\boldsymbol{H}_{\mathbf{e}} > 0$.

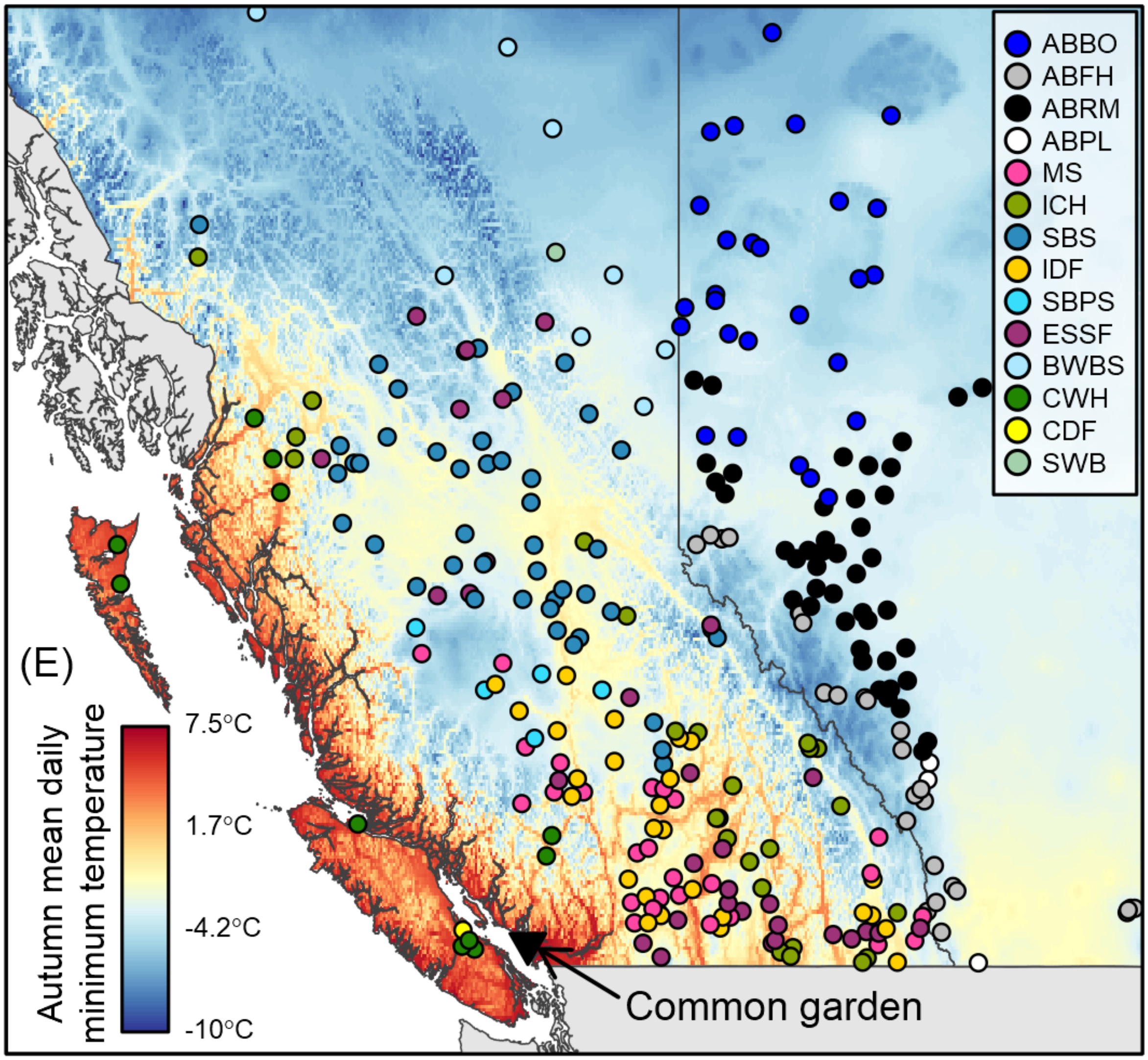

79
80 **Figure S13: Biogeoclimatic zones (British Columbia) and natural regions (Alberta) of each sampled provenance.**

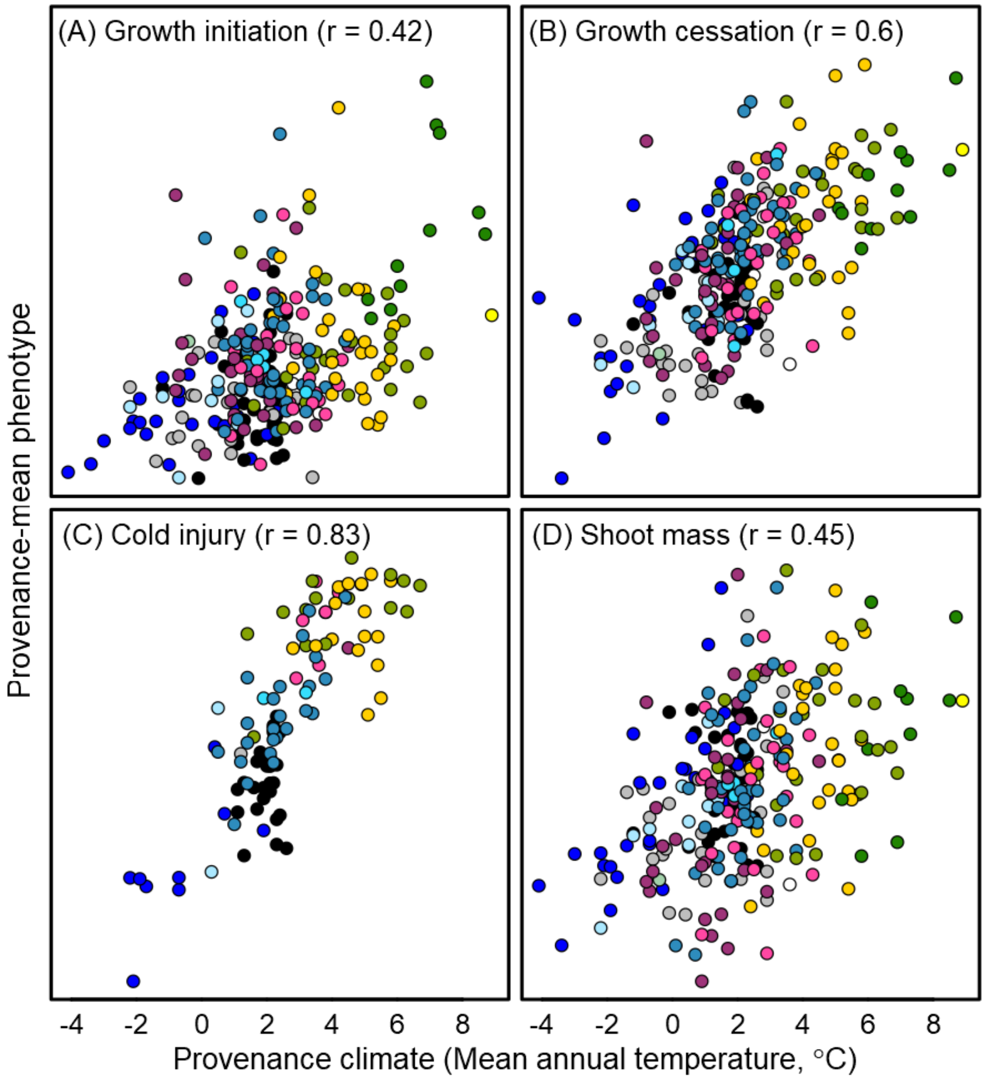

Figure S14: Phenotypic clines of four traits in lodgepole pine seedlings grown in the Vancouver common garden, colour themed by biogeoclimatic zone (British Columbia) and natural region (Alberta). See Figure S13 for map and color schemes.

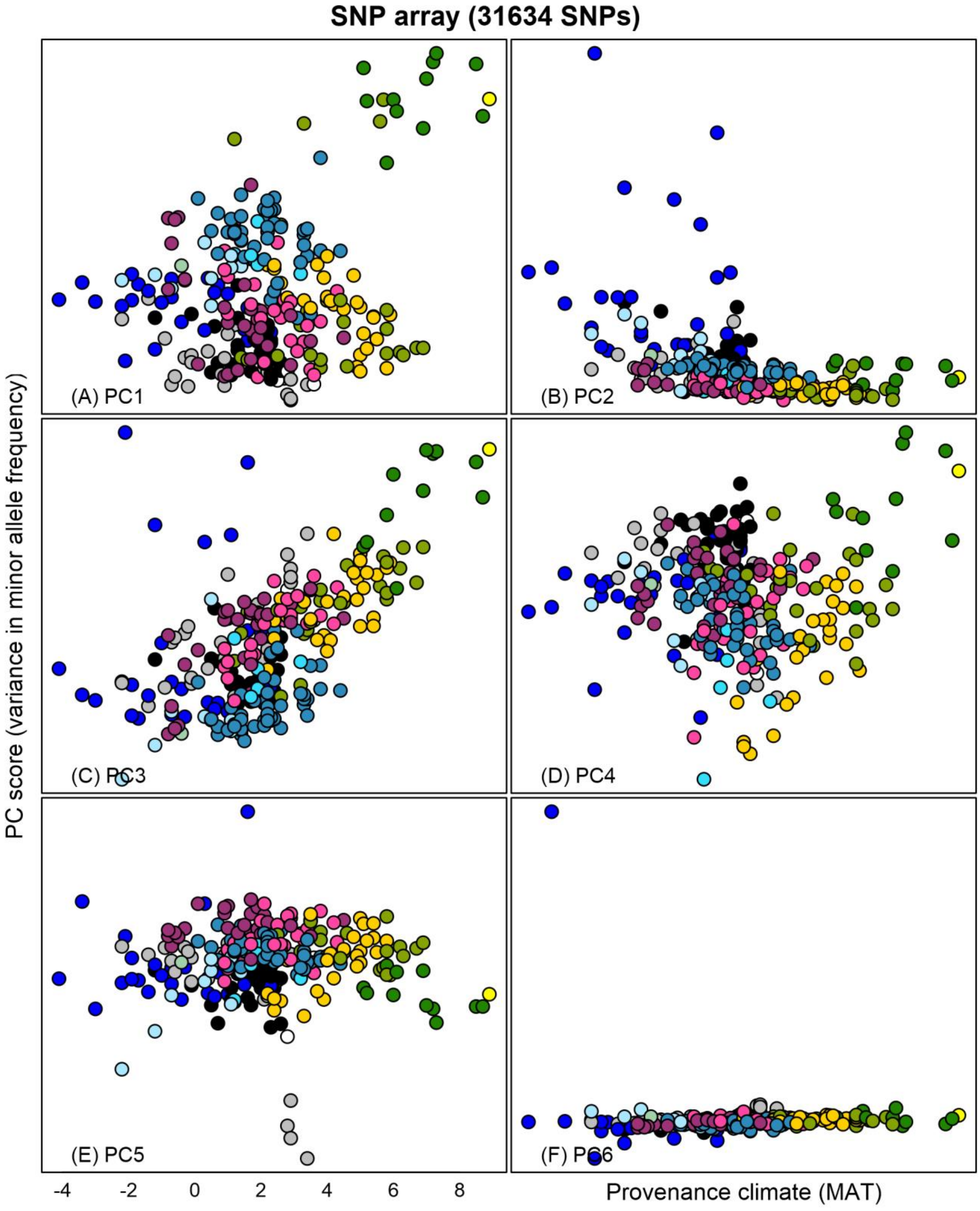

Figure S15. First six principal components of *z*-standardized population-mean minor allele frequencies in the full SNP array (excluding the control set), plotted against mean annual temperature. Populations are colour themed by biogeoclimatic zone (British Columbia) and natural region (Alberta) of their provenance. See Figure S13 for map and color scheme.

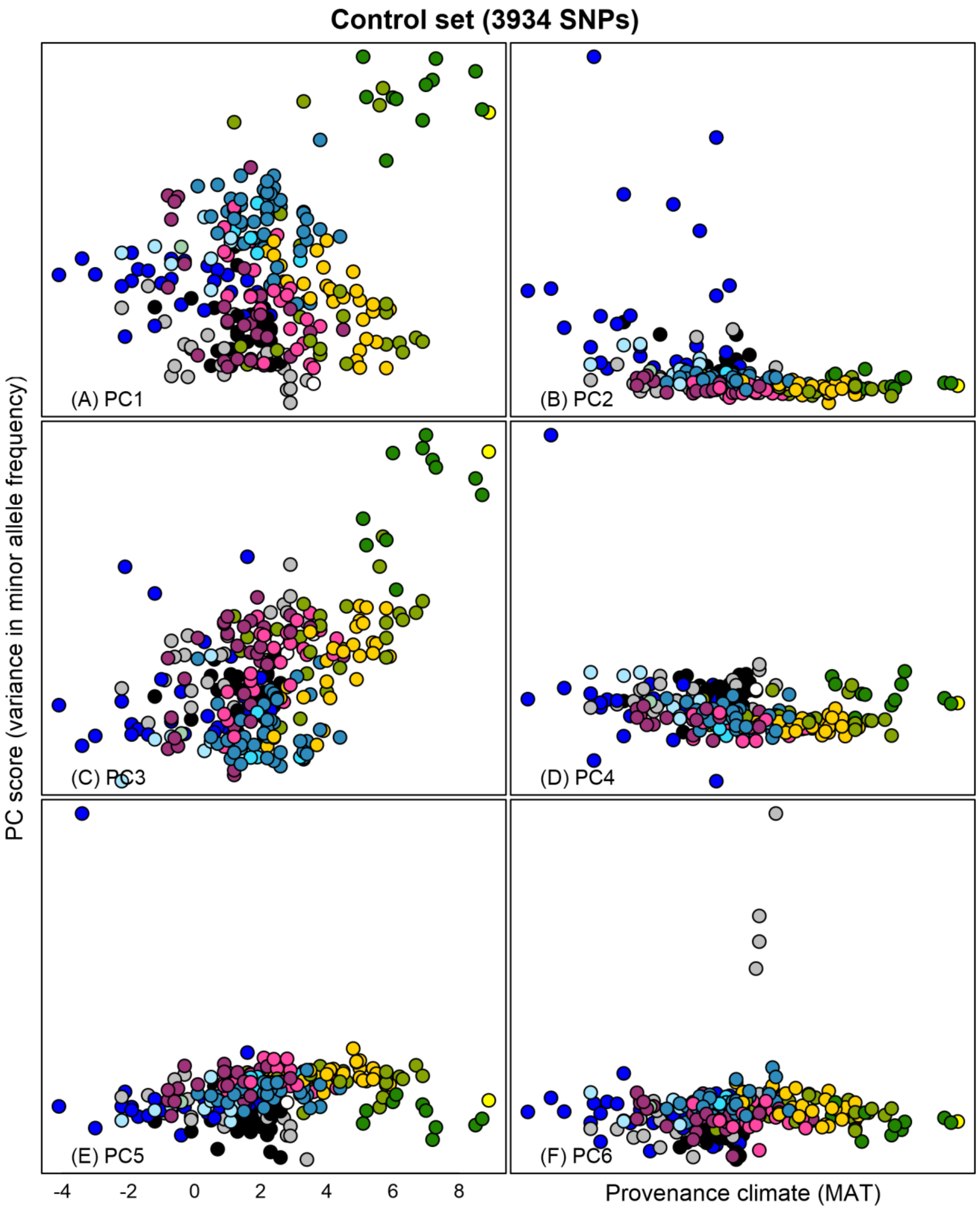

Figure S16. First six principal components of *z*-standardized population-mean minor allele frequencies in the control set, plotted against mean annual temperature. Populations are colour themed by the biogeoclimatic zone (British Columbia) and natural region (Alberta) of their provenance. See Figure S13 for map and color scheme.

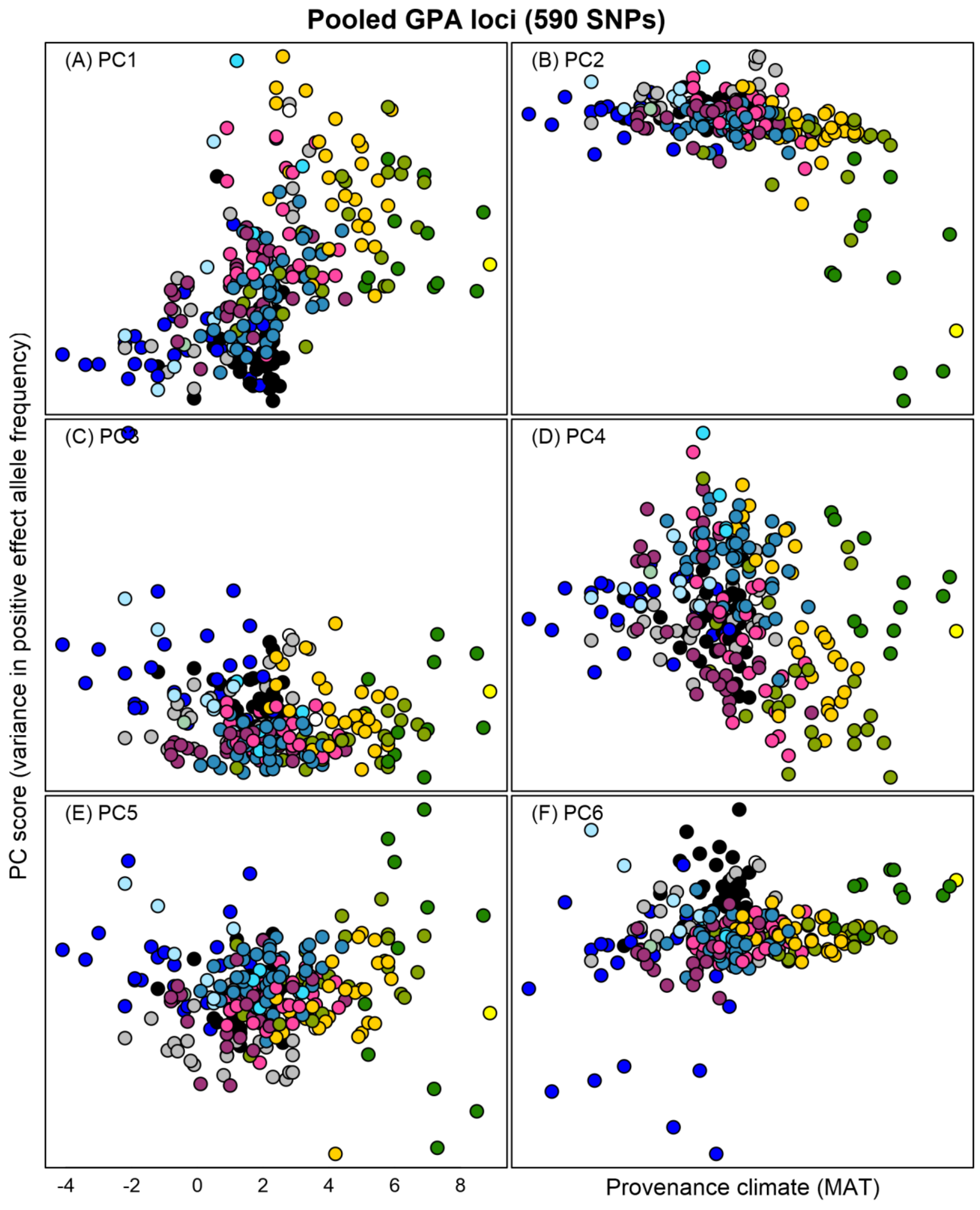

Figure S17. First six principal components of z-standardized population-mean positive-effect allele frequencies in the pooled GWAS loci for all four common garden traits, plotted against mean annual temperature. Populations are colour themed by the biogeoclimatic zone (British Columbia) and natural region (Alberta) of their provenance. See Figure S13 for map and color scheme.

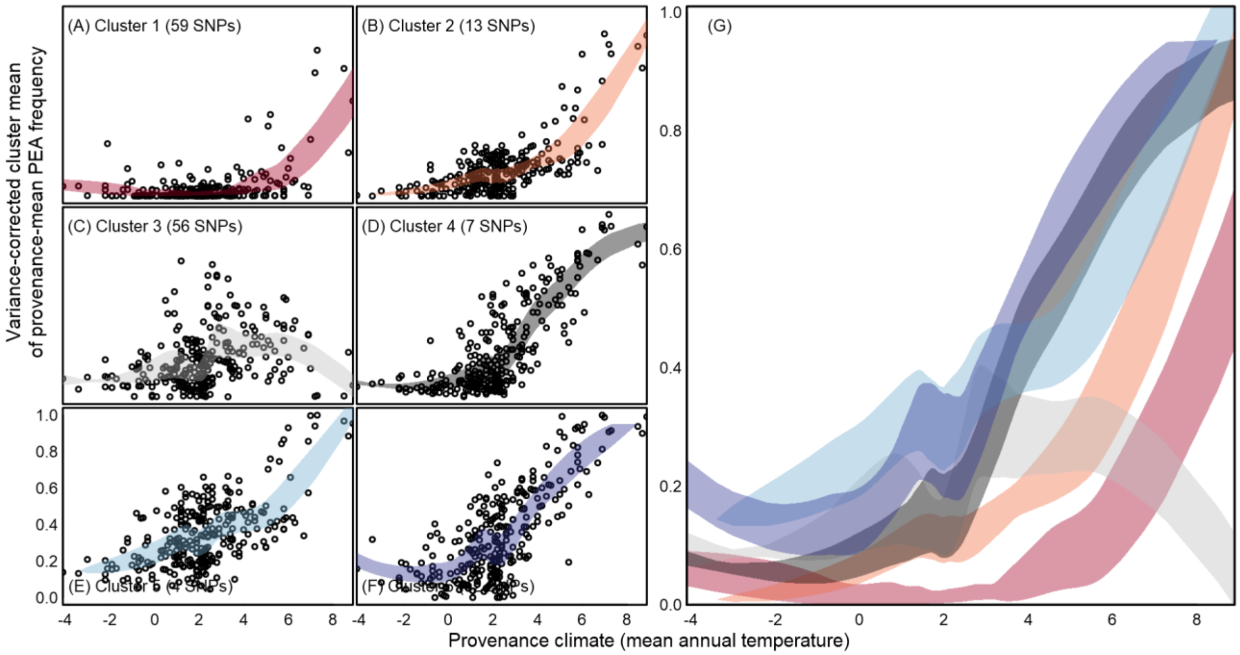

Figure S18. As in Figure 5, but for timing of growth initiation instead of autumn cold injury

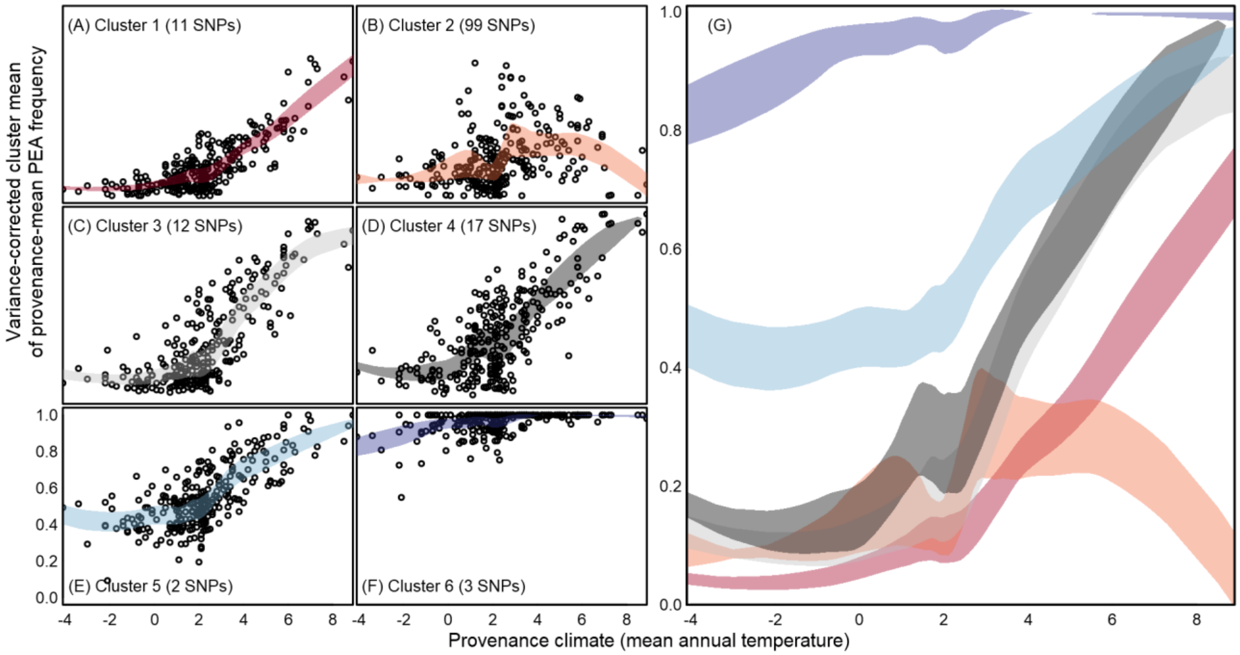

Figure S19. As in Figure 5, but for timing of growth cessation instead of autumn cold injury

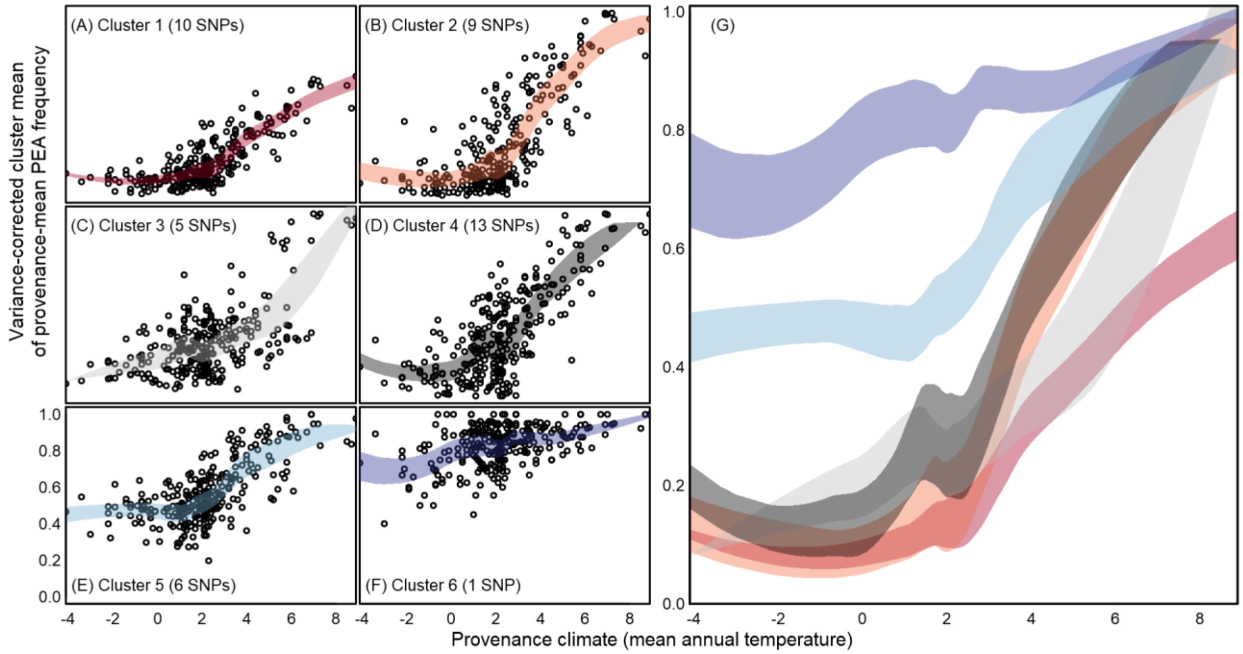

Figure S20. As in Figure 5, but for shoot mass instead of autumn cold injury
